# A randomized controlled trial comparing non-steroidal anti-inflammatory and fusion protein inhibitors singly and in combination on the histopathology of bovine respiratory syncytial virus infection

**DOI:** 10.1371/journal.pone.0252455

**Published:** 2021-06-10

**Authors:** Francisco R. Carvallo Chaigneau, Paul Walsh, Maxim Lebedev, Victoria Mutua, Heather McEligot, Heejung Bang, Laurel J. Gershwin

**Affiliations:** 1 Division of Veterinary Pathology, Department of Biomedical Sciences & Pathobiology Virginia Tech, Blacksburg, VA, United States of America; 2 Dept. of Pathology, Microbiology & Immunology, School of Veterinary Medicine, University of California, Davis, Davis, CA, United States of America; 3 Pediatric Emergency Medicine, The Sutter Medical Center Sacramento, Sacramento, CA, United States of America; 4 Division of Biostatistics, Department of Public Health Sciences, University of California Davis, Davis, CA, United States of America; Wageningen Bioveterinary Research, NETHERLANDS

## Abstract

Bovine respiratory syncytial virus (RSV) has substantial morbidity in young calves, and closely parallels human RSV in infants. We performed a randomized controlled trial in five to six-week-old Holstein calves (*Bos taurus*). comparing fusion protein inhibitor (FPI) and non-steroidal anti-inflammatory drug (NSAID) singly and in combination at three and five days after experimental BRSV infection. Thirty-six calves received one of six treatments; Ibuprofen started on day 3, Ibuprofen started on day 5, FPI started on day 5, FPI and Ibuprofen started on day 3, FPI and Ibuprofen started on day 5, or placebo. We have previously reported significant clinical benefits when combined FPI and NSAID treatment was started at three and five days after bovine RSV infection. Necropsy was performed on Day 10 following infection and hematoxylin and eosin staining was performed on sections from each lobe. Histology was described using a four-point scale. We performed canonical discrimination analysis (CDA) to determine the structural level where differences between treatments occurred and mixed effects regression to estimate effect sizes. Separation from placebo was maximal for dual therapy at the levels of the alveolus, septum, and bronchus in CDA. We found that the clinical benefits of combined FPI and NSAID treatment of BRSV extend at least partially from histopathological changes in the lung when treatment was started three days after infection. We found decreased lung injury when ibuprofen was started as monotherapy on day 3, but not day 5 following infection. Combined therapy with both an FPI and ibuprofen was always better than ibuprofen alone. We did not prove that the clinical benefits seen starting FPI and ibuprofen five days after infection can be solely explained by histopathological differences as identified on H&E staining.

## Introduction

Bovine respiratory syncytial virus (BRSV) has substantial morbidity and mortality alone or as the inciting agent in bovine respiratory disease complex in young calves [1, 2]. BRSV is costly both in direct and indirect productivity losses. Infected calves who survive often develop recurring cough and poor weight gain in response to dusty environments, due to development of an enhanced hypersensitivity response to environmental antigens [1, 3].

Human respiratory syncytial virus (HRSV) is a closely related virus that is the most common inciting agent in bronchiolitis. Bronchiolitis is a major cause of hospital admission in winter and spring in infants [4]. HRSV also precedes or triggers recurrent antigen induced wheezing following initial infection in 48% of infants [5]. HRSV in infants follows a similar clinical trajectory to BRSV in calves, has similar lung ultrasound manifestations, and demonstrate similar immunological characteristics [6–9].

Some of the BRSV lung injury stems, directly and indirectly, from the animals own immune responses [3, 10, 11]. Both this acute inflammatory response, and the formation of antibodies to bystander antigens (leading to later wheezing) could potentially be blunted with non-steroidal anti-inflammatory drugs [12–14]. These apparent benefits of NSAID treatment come at the cost of increased viral reproduction and raise the potential for harm [12, 15, 16].

Antiviral drugs for RSV are in development. Structurally almost identical antivirals targeting the RSV fusion protein (FPI) have shown promise in both humans and cattle [17, 18]. FPI are most effective if started prior to, or at the time of inoculation with BRSV [13]. If initiated after inoculation, current evidence shows histological benefit when antiviral FPI treatment is started up to 3 days following inoculation with BRSV [18]. Three days following infection would translate to a very short therapeutic window between the onset of symptoms and time to initiate treatment and an important clinical question is: can this window be extended?

Dual drug therapy with both an FPI and NSAID has the potential to mitigate the increase in viral shedding caused by using NSAIDs alone [13, 19]. Combining the immunomodulating effects of an NSAID with an antiviral drug might be expected to lead to more benefit than using either alone. We have previously reported a randomized controlled trial to test this hypothesis. We found that clinical outcomes were improved when combined antiviral and NSAID treatment were started at 3- and 5-days post BRSV inoculation [16].

Here we try to answer two questions. Do the apparent clinical benefits of combined antiviral and NSAID treatment of BRSV extend from histopathological changes in the lung? And if they do, how soon after inoculation must treatment be started to prevent histological damage to the lung?

## Materials and methods

We have previously reported the clinical findings from the parent study [16]. We randomized 36 Holstein calves aged five to six weeks to receive placebo; GS-56193 (previously designated GS1); a fusion protein inhibitor at 600 mg twice daily, ibuprofen; an NSAID at 10 mg/kg three times daily, or both. We randomized calves to one of six treatment groups using minimization based on maternal antibody titer and weight.

Minimization ensured balance at the individual level for maternal antibody titer and calf weight on the day of randomization and provided the best chance for attaining balance at the group level on all other covariates [20]. The techniques used to induce experimental BRSV infection [6, 12, 21] and the specific experimental protocol used for this study are detailed elsewhere [16]. Because of the workload involved we performed the experiment in three replicates of 12 bovine calves each. Each replicate had two calves from each of the six treatment groups. All medications and identical appearing placebos were administered orally. Drug allocation was equal across treatment groups and replicates. Treatment groups are shown in Table 1.

**Table 1:** All calves received placebo from day 0 until the first day of their active treatment.

Treatment	Days after infection	Number per batch/replicate	Total N
Ibuprofen	3–10	2	6
Ibuprofen	5–10	2	6
Placebo	0–10	2	6
FPI	5–10	2	6
FPI + ibuprofen	5–10	2	6
FPI + ibuprofen	3–10	2	6

This was to blind the research team to treatment allocation.

Animals were scheduled for euthanasia and necropsy on Day 10 following inoculation using pentobarbital overdose administered via the jugular vein. Criteria for early euthanasia were temperature over 40°C (normal range from 37.8 to 39.2°C) with depression, dyspnea causing open mouth breathing, inability to stand, and inability to drink from a bottle. Following euthanasia, a necropsy was performed. We took bronchial and lung swabs for bacterial culture and polymerase chain reaction (PCR) testing for Mycoplasma, BRSV, bovine herpes virus, and bovine viral diarrhea virus.

The UC Davis Institutional Animal Care and Use Committee (authorization number 19313) approved this study.

### Estimation of extent of pneumonia

The percentage lung consolidation for each lobe was estimated semi-quantitively by inspection by an experienced board-certified veterinary pathologist (FRC).

### Histological scoring

**Fig 1:**
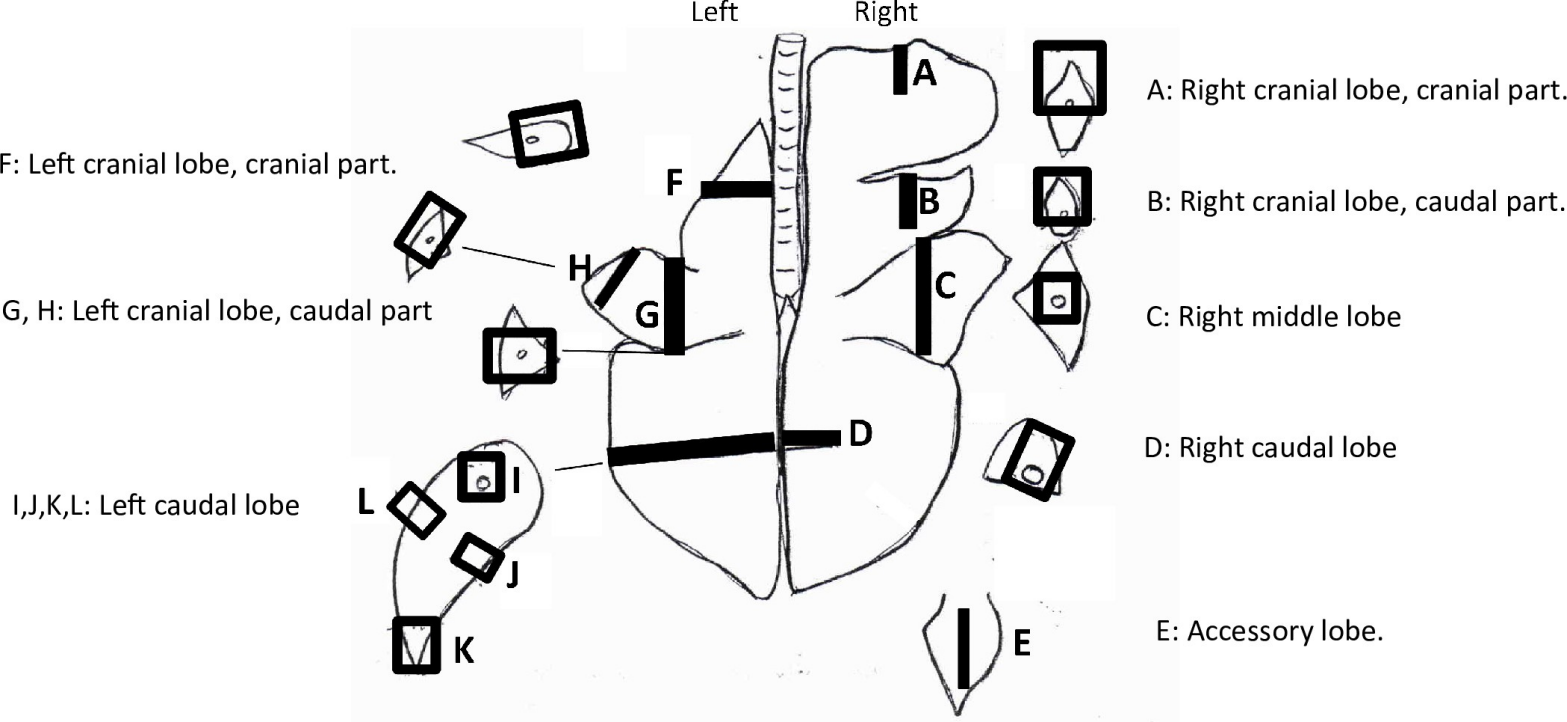
Anatomical map indicating where slides were sectioned from in the lungs. These letters are carried forward in the subsequent graphs.

The left lung was perfused with formalin at 15 cm water pressure. Sections of the right lung were collected and fixed for at least 24 hours in 10% formalin, as shown on the schema in Fig 1. The letter used to identify the location from where the slide was taken is retained throughout the subsequent analysis. Hematoxylin and eosin (H&E) staining was performed. A veterinary pathologist (FRC) used a 4-point ordinal scale to describe the findings as shown in Table 2. Histological scoring was required because unlike organs such as the liver where changes are homogeneous making side-by-side photomicrographs useful, lung pathology does not lend itself to such analysis. Each case has at least 14 slides, some with lesions, some without lesions. Within slides there will be normal and abnormal areas, one lobule can have severe lesions, the other none (due to lack of collateral ventilation). Each structural level (e.g. alveolus, bronchiole, pleura) will react differently to infection and even within each level there are different variables (inflammation, fibrin, hemorrhages, etc.) and not just one that can be compared side by side. We summed these scores to provide a quantitative score of lung histology as described elsewhere [22]. We did not measure intra-rater reliability.

**Table 2:** Specific histological findings sought and scored for each structure on each slide.

Pleura	Alveolus	Septum	Interstitium	Bronchiole	Bronchus
Expansion	Atelectasis	Arteritis	Lymphoid nodules	Epithelial transmigration	Deciliation
Expansion edema	Edema	Edema	Monocytic thickening of interstitium	Fibrinous exudates	Epithelial transmigration
Fibrosis	Fibrinous exudate	Expansion	PMN/Eosinophilic thickening of interstitium	Inclusion bodies	Inclusion bodies
Lymphatic dilation	Granulomas	Fibrin	Thrombosis	Neutrophilic exudates	Intraepithelial pustules
Lymphatic dilation edema	Hemorrhages	Fibrosis	Vasculitis	Bronchiolitis obliterans	Lymphoid nodules
Monocytic infiltrates	Hyperplasia type II pneumocytes	Monocytic infiltrates		Peribronchiolar lymphoid nodules	Monocyte submucosal infiltrates
Neutrophilic infiltrates	Neutrophilic exudates	Neutrophilic infiltrates		Peribronchiolar monocytic infiltrates	Neutrophilic exudates
Pleocellular infiltrates		Pleocellular infiltrates		Necrosis of epithelial cells	
Thickening	Syncytial cells				
	Lytic necrosis multi focal				
	Necrosis				

Items were scored as 0 to 3 for none, mild, moderate, or severe. The full grading system is provided in S1 File “Histological grading of pulmonary lesions”.

### Clinical outcomes

The details of the clinical outcomes are described elsewhere [16]. Briefly, we performed daily physical exams and used a previously published scoring tool each day of the experiment. Higher clinical scores indicated more severe disease. For this report we calculated quartile within which each calf fell for peak and mean clinical score over the duration of the experiment. Higher clinical scores indicated more severe disease. For this report we calculated quartile within which each calf fell for peak and mean clinical score over the duration of the experiment and used those quartiles in our matrix plots. The other variables in the matrix plots were scored 0 to 3.

### Viral load

The details of how viral load was measured using nasal swabs has been described elsewhere [16]. Briefly, we took nasal swabs every other day and performed PCR using primers specific to bovine RSV nucleoprotein: forward primer GCAATGCTGCAGGACTAGGTATAAT and reverse ACACTGTAATTGATGACCCCATTC. The RT-PCR thermocycling program consisted of one cycle of 50°C for 2 minutes and 95° C for 10 minutes, followed by 40 cycles of 95°C for 15 seconds and 60°C for 1 minute. Standard curve fitting and virus quantification was performed using ViiA7 software (ThermoFisher Scientific, Waltman, MA). We calculated the quartile within which each calf fell for peak and mean viral load and used these quartiles in our matrix plots. The other variables in the matrix plots were scored 0 to 3.

### Data analysis

In the first part of the analyses, we graphed the raw data using matrix plots. The first matrix plot shows the percentage consolidation for each lobe for each calf. The other matrix plots: one for each structure e.g. alveolus, pleura, etc., show the histological severity for each finding. We calculated the overall score as the sum of the histology scores (0–3) for each finding for each slide added to 2x (sum of percentage consolidation of each lobe). We fitted a mixed effects regression with this score as the outcome variable, and treatment groups and variables used during minimization as independent variables at the fixed effects, and replicate as the random effects level. We performed the same analysis separately for histological changes and the sum of percentage consolidation for each lobe as the outcome. We compared regression models using likelihood ratios. Fit characteristics for the final models were assessed graphically. We used restricted maximum likelihood estimation recommended by some for smaller sample sizes [23].

Our analytical approach reflected the practicalities inherent in our experiment. While ANOVA of histological scores is widely used to demonstrate differences in treatment outcomes [6, 24–29]. The workload involved in 36 calves required that we batch our experiment into three replicates. Multilevel regression addresses the fact that when studies are divided across study sites, time, or batches there will inevitably be variability between each site, time- period or batch [30]. Such variability exists even when every effort is made to keep experimental conditions the same. This was especially true in this experiment; we used out-bred farm animals from different birth cohorts and the weather was different for each batch.

Using the data minimization method of randomization increased the statistical power of the experiment. However, despite data minimization being described as “the platinum standard” of randomization [20], data minimization is a form of stratified randomization, and having calves from each treatment group in each replicate was a form of blocking [20, 31]. Blocking and stratification can break the independence between treatment groups complicating the final analysis [32]. Multilevel regression permits adjustment for factors used in blocked and/or stratified randomization.

Multilevel regression also allows increased efficiency by including variables that affect outcome but are not of interest to the researcher-for example calf weight at the time of randomization or maternal antibody titer. Despite inaccurate widespread perceptions to the contrary, such (prespecified) covariate adjustment is the optimal method for analyzing randomized controlled trials [33–36]. After estimating with various model specifications, we dropped ‘maternal antibody titer’ and retained ‘calf weight at randomization’ in the fixed effects part. The multilevel regression models had better fit characteristics than the simple regression both with and without an indicator variable for replicate (batch).

We compared each of the treatment groups with each other using both individual analyses for each treatment pair, and together in a single model with pairwise comparison. Apart from pairwise comparison within the primary model using placebo as referent we did to adjust for multiple comparisons.

We used mixed effects regression with viral shedding as the dependent variable with levels for replicate and calf and spline of day and drug treatment group in the fixed parts. We included the infecting dose of bovine RSV in the fixed effects and random effects parts of the model. We followed this with pairwise comparison. This allowed us to compare viral shedding with the histological scores for each treatment group. We took a similar approach to clinical score.

We recognize both the necessity for, and the limitations of scoring systems for histology. The scoring system reduces the number of histological variables from hundreds to one. In doing so our scoring system masks the difficulties inherent in analyzing data where the number of variables far exceeds the number of subjects. Therefore, in the third part of the analyses we performed discriminant analysis to complement our mixed effects model. Discriminant analysis is also a variable reduction technique but less arbitrary than scoring systems. Canonical discriminant analysis is a supervised form of discriminant analysis suited to descriptive (as distinct from predictive) analysis [37]. Canonical discriminant analysis, in contrast to other forms of discriminant analysis, does not assume the hypothesized groups are empirically distinct and exclusive, rather the hypothesized groups are evaluated on the discriminating variables after the functions are extracted to determine if the groups are distinguishable on the basis of the information the functions contain [38].

We had between one and four representative slides from each lobe resulting in 48 to 192 histological findings for each anatomical lobe, but only six animals in each treatment group. Canonical discriminant analysis reduces the variables into a small number of discriminant functions. Each of these discriminant functions is a linear combination of the discriminating variables that provide maximal separation between the grouping variable-in this case, treatment [39]. We plotted the two functions which explained most variance on the x-and y-axes to create a score plot. After this we labeled each calf’s data point to determine if individual treatment groups represented distinct histological entities or if they overlapped. Overlap would imply no difference in histological outcomes by treatment. Collinear variables and variables with the same value regardless of the treatment group were dropped from the canonical discriminant analysis.

## Results

Two of the 36 calves required early euthanasia, one in the placebo and one in the ibuprofen alone at 3 days group, all were necropsied. Histopathological scores were highest in calves receiving placebo throughout and those who started monotherapy with ibuprofen after 5 days. Viral shedding was highest in those receiving ibuprofen monotherapy. Both histopathological scores and viral shedding were markedly reduced when dual therapy with ibuprofen and FPI was started three days following inoculation.

**Fig 2:**
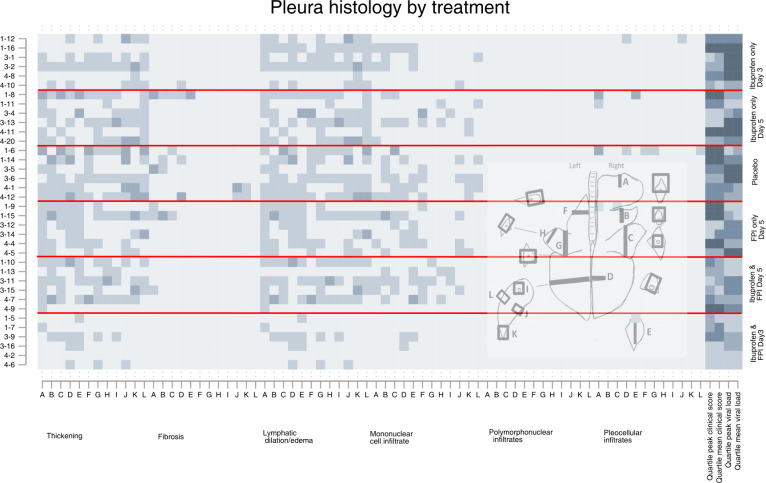
Pleural histology findings by drug treatment group. Each row represents an individual calf, each letter represents a slide, the x-axis colors indicate a different specific histological finding. The shading of each pixel represents the severity of the finding on a scale of 0 (light) to 3 (darker). On the y-axis the first number represents the batch/replicate number, the number following the dash the ear tag for the individual calf. The horizontal red lines divide the treatment groups. The last four columns represent quartiles (first lightest, fourth darkest) of mean peak and clinical scores and mean peak viral loads measured by nasal swabs.

**Fig 3:**
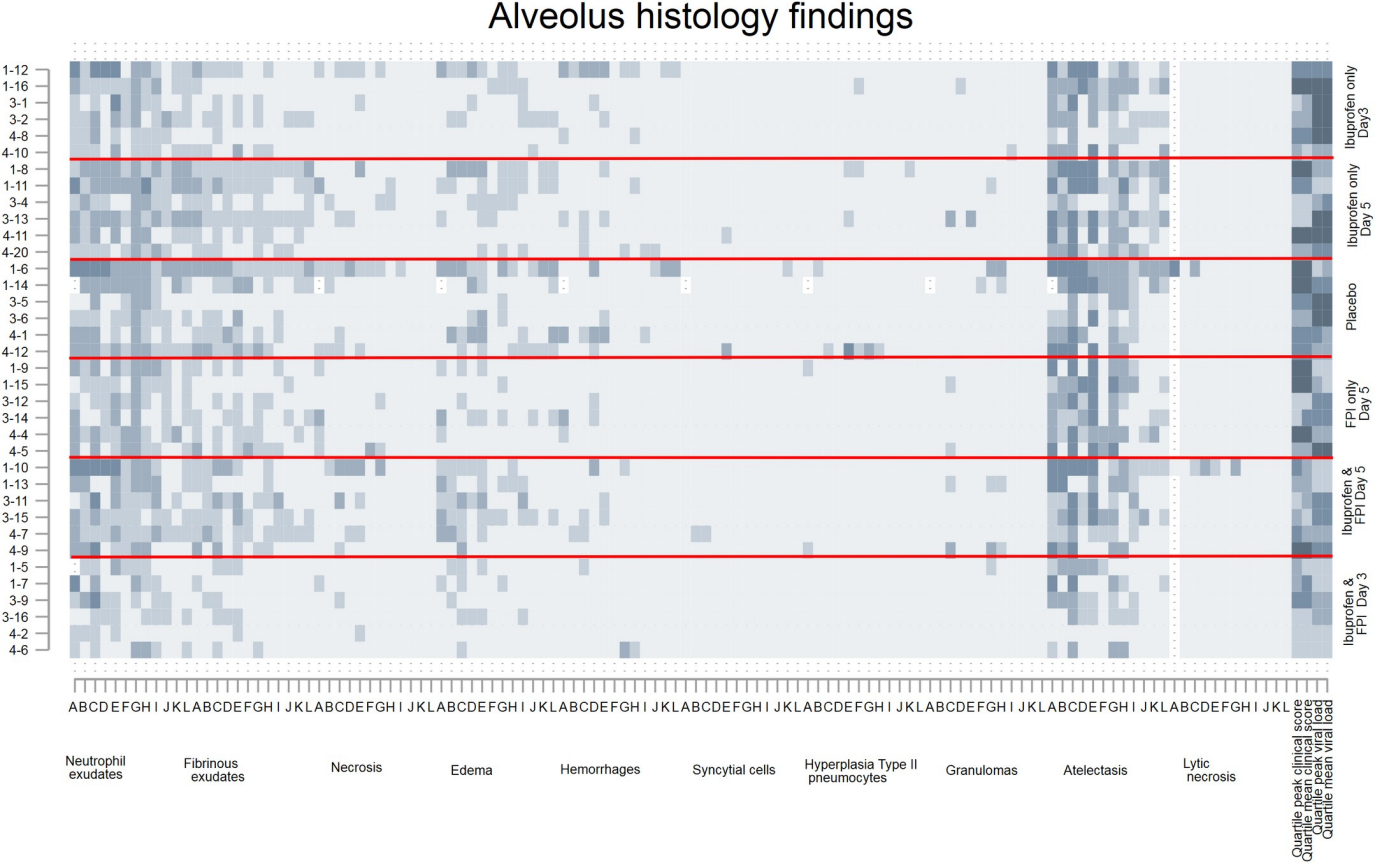
Alveolus histology findings by drug treatment group. Each row represents an individual calf, each letter represents a slide, the x-axis colors indicate a different specific histological finding. The shading of each pixel represents the severity of the finding on a scale of 0 (light) to 3 (darker). On the y-axis the first number represents the batch/replicate number, the number following the dash the ear tag for the individual calf. The horizontal red lines divide the treatment groups. The last four columns represent quartiles (first lightest, fourth darkest) of mean peak and clinical scores and mean peak viral loads measured by nasal swabs.

**Fig 4:**
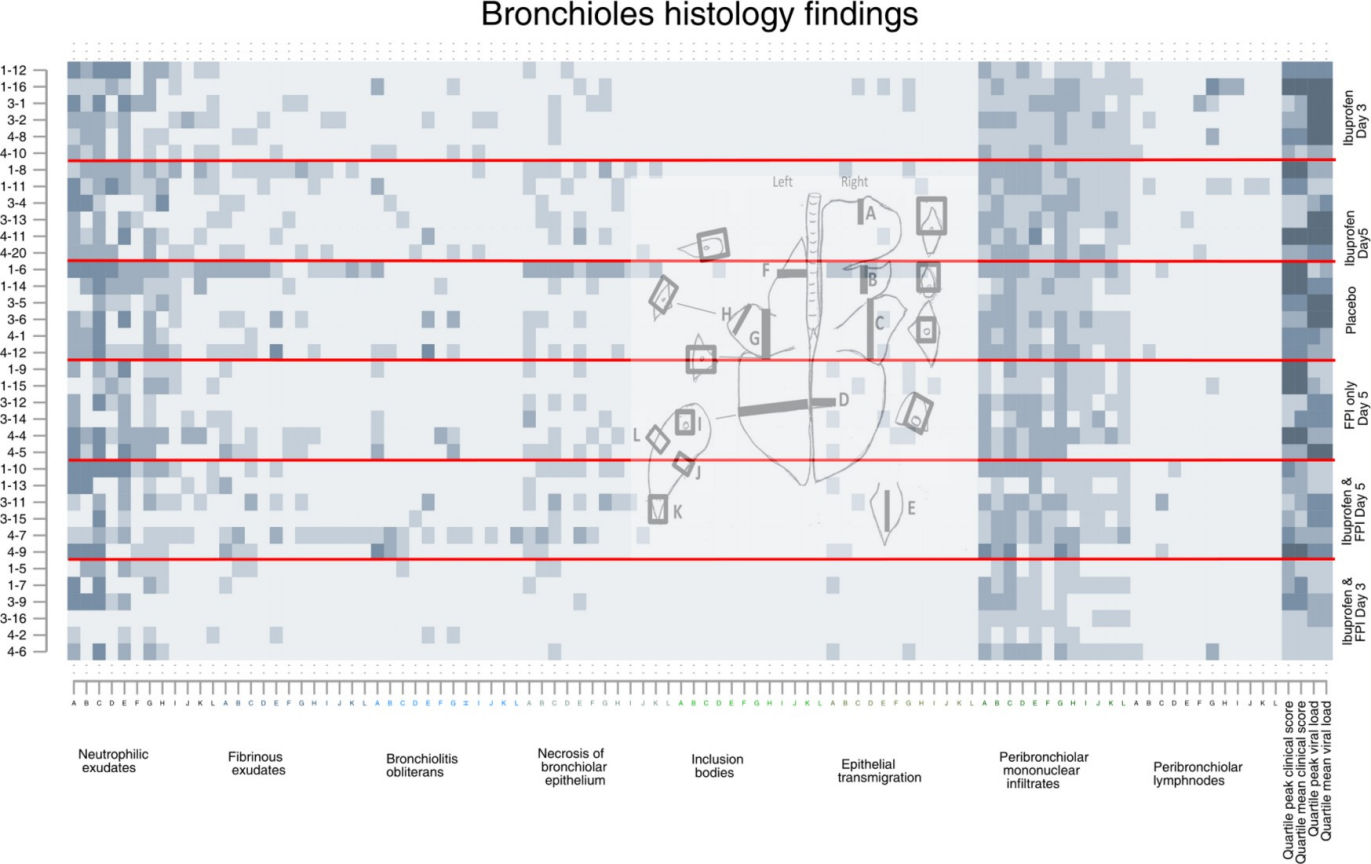
Bronchiole histology findings by drug treatment group. Each row represents an individual calf, each letter represents a slide, the x-axis colors indicate a different specific histological finding. The shading of each pixel represents the severity of the finding on a scale of 0 (light) to 3 (darker). On the y-axis the first number represents the batch/replicate number, the number following the dash the ear tag for the individual calf. The horizontal red lines divide the treatment groups. The last four columns represent quartiles (first lightest, fourth darkest) of mean peak and clinical scores and mean peak viral loads measured by nasal swabs.

**Fig 5:**
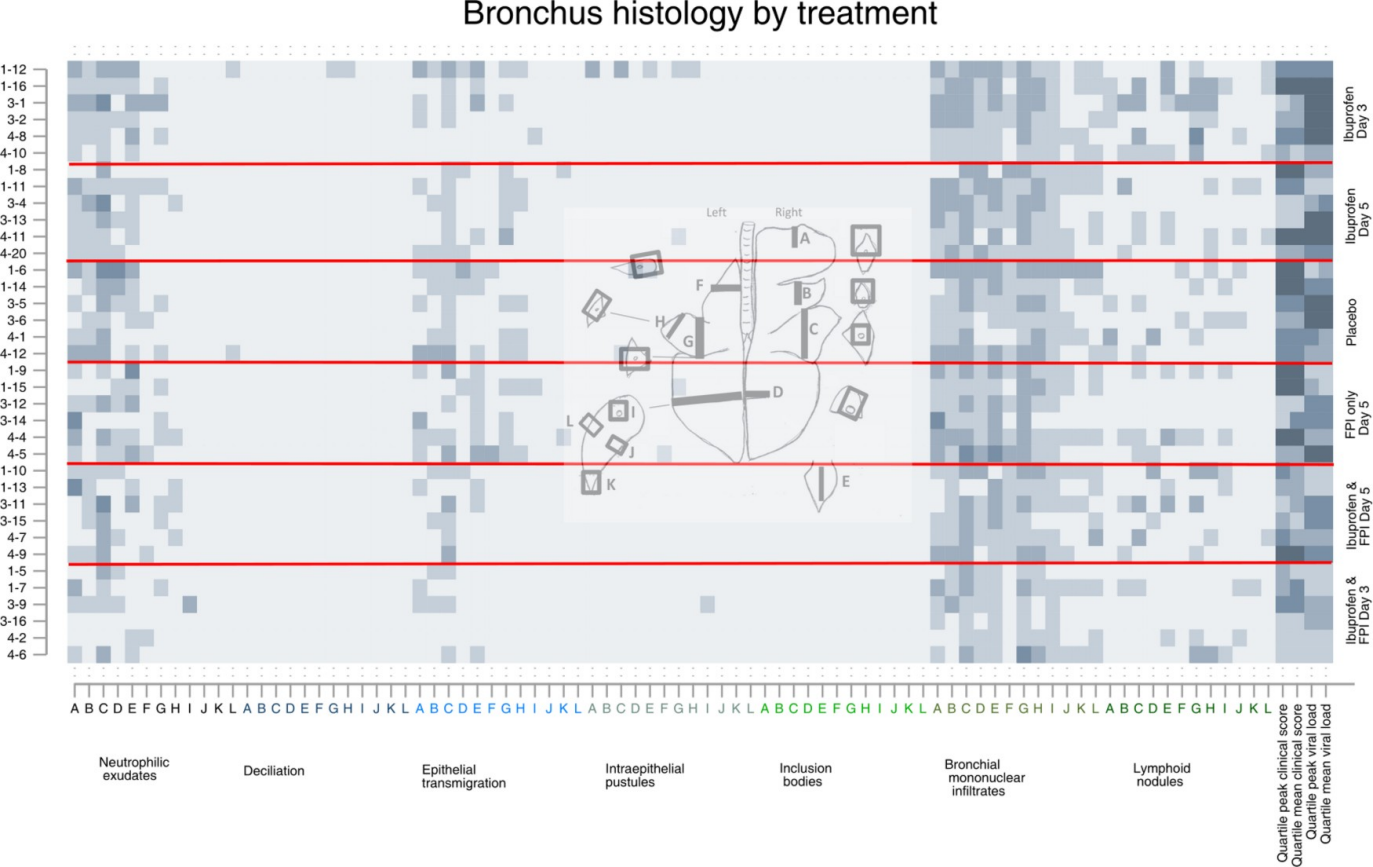
Bronchus histology findings by drug treatment group. Each row represents an individual calf, each letter represents a slide, the x-axis colors indicate a different specific histological finding. The shading of each pixel represents the severity of the finding on a scale of 0 (light) to 3 (darker). On the y-axis the first number represents the batch/replicate number, the number following the dash the ear tag for the individual calf. The horizontal red lines divide the treatment groups. The last four columns represent quartiles (first lightest, fourth darkest) of mean peak and clinical scores and mean peak viral loads measured by nasal swabs.

**Fig 6:**
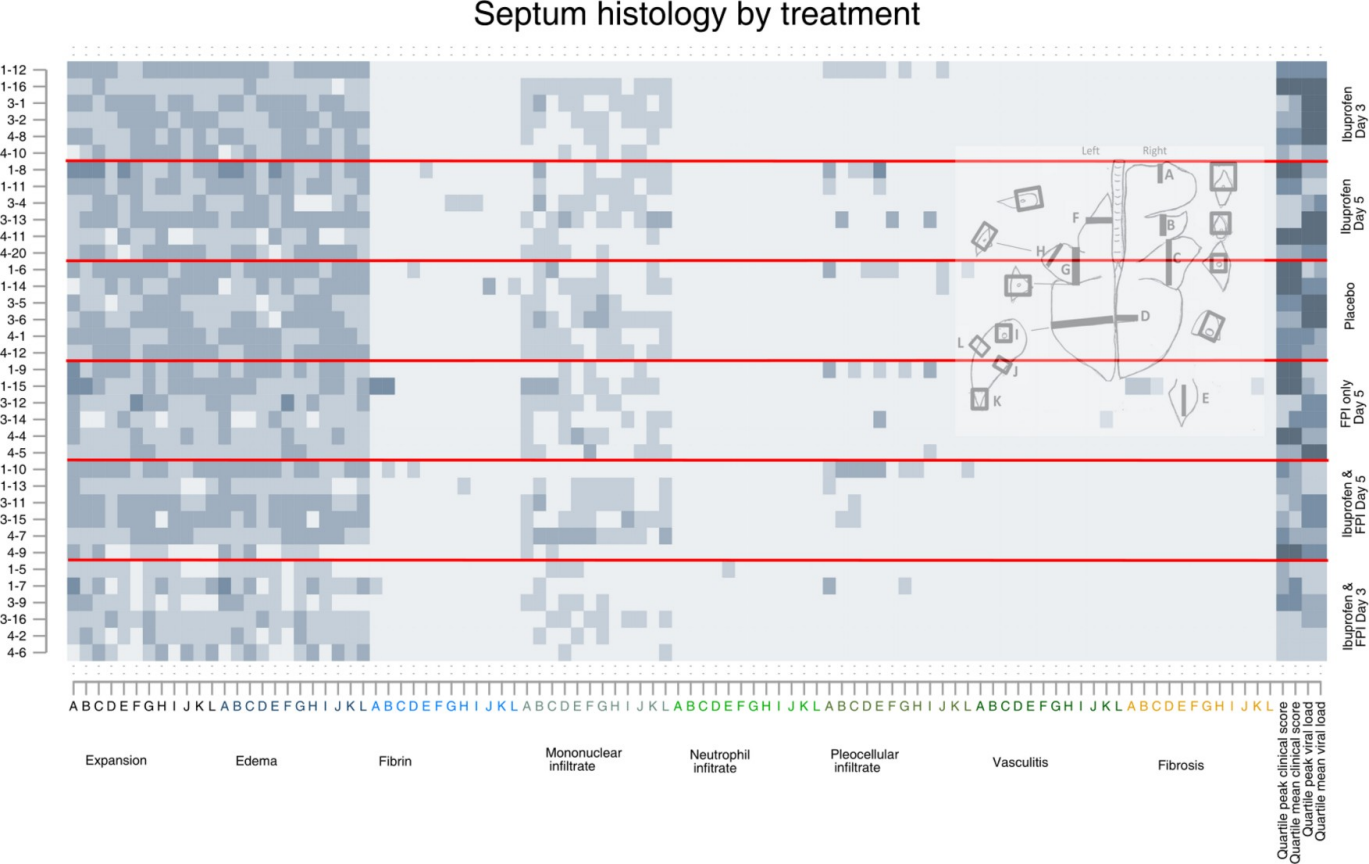
Septum histology findings by drug treatment group. Each row represents an individual calf, each letter represents a slide, the x-axis colors indicate a different specific histological finding. The shading of each pixel represents the severity of the finding on a scale of 0 (light) to 3 (darker). On the y-axis the first number represents the batch/replicate number, the number following the dash the ear tag for the individual calf. The horizontal red lines divide the treatment groups. The last four columns represent quartiles (first lightest, fourth darkest) of mean peak and clinical scores and mean peak viral loads measured by nasal swabs.

**Fig 7:**
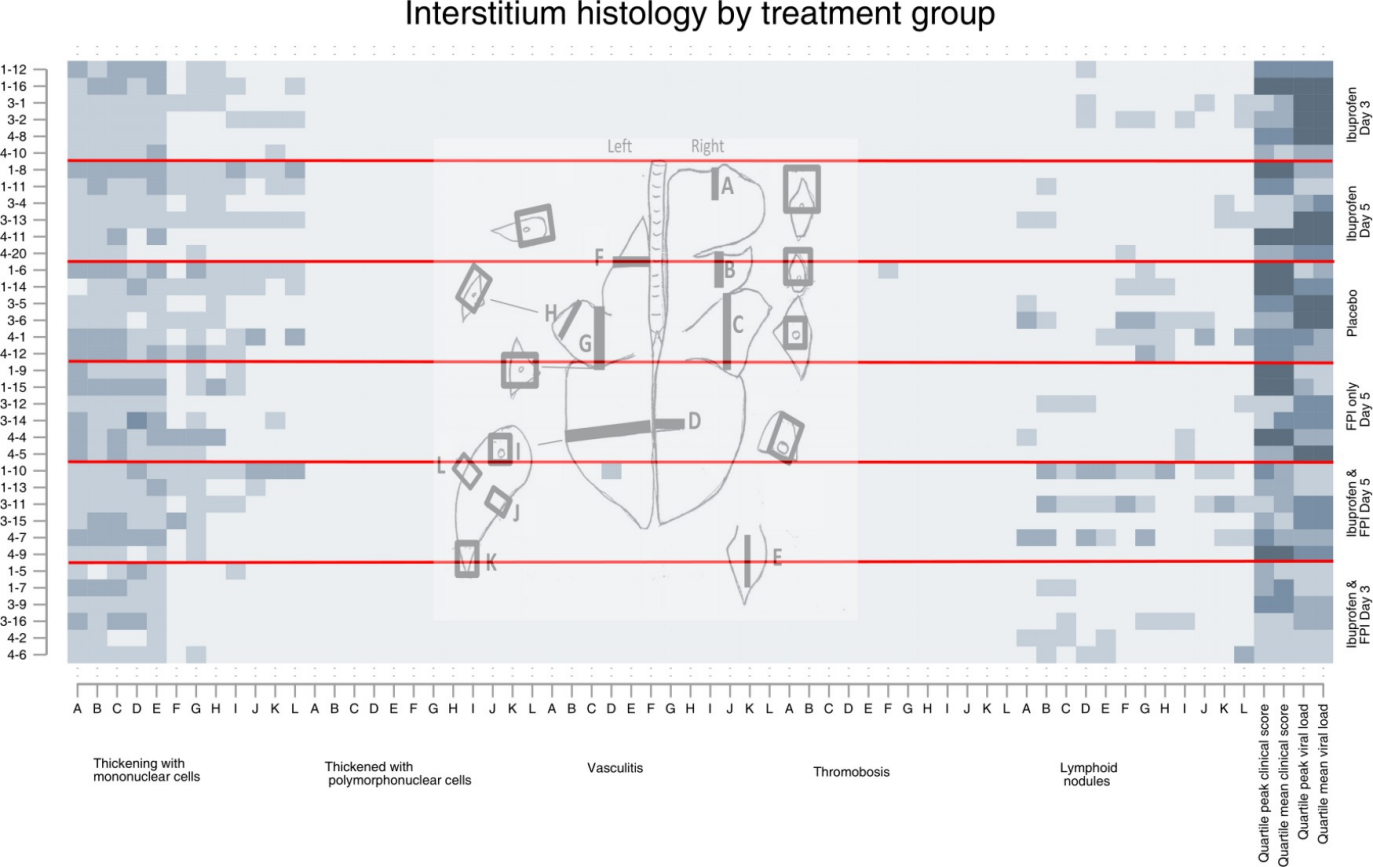
Interstitium histology findings by drug treatment group. Each row represents an individual calf, each letter represents a slide, the x-axis colors indicate a different specific histological finding. The shading of each pixel represents the severity of the finding on a scale of 0 (light) to 3 (darker). On the y-axis the first number represents the batch/replicate number, the number following the dash the ear tag for the individual calf. The horizontal red lines divide the treatment groups. The last four columns represent quartiles (first lightest, fourth darkest) of mean peak and clinical scores and mean peak viral loads measured by nasal swabs.

**Fig 8:**
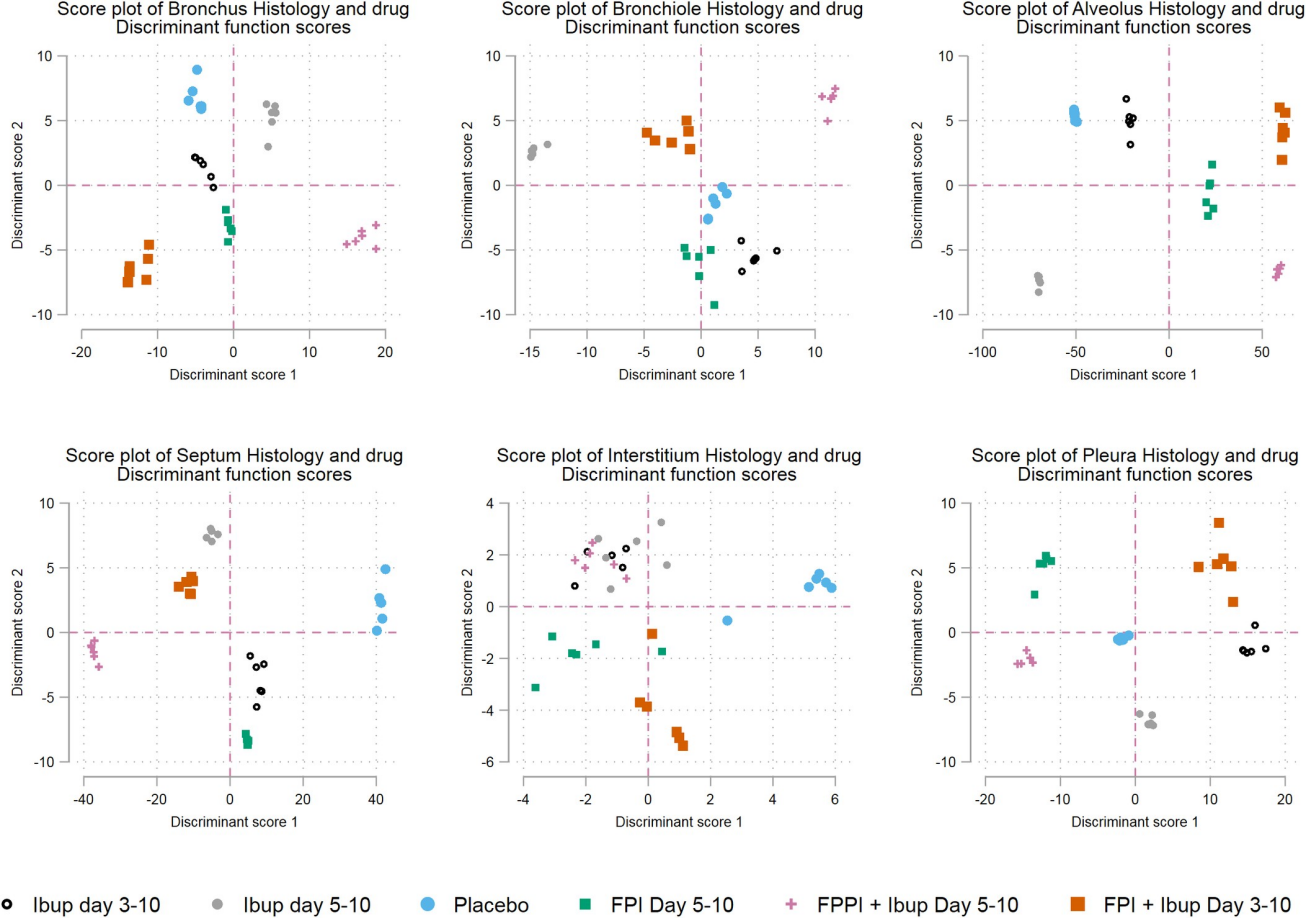
Canonical discriminant analysis for histological findings at the structural level. FPI; fusion protein inhibitor, Ibup; Ibuprofen.

**Fig 9:**
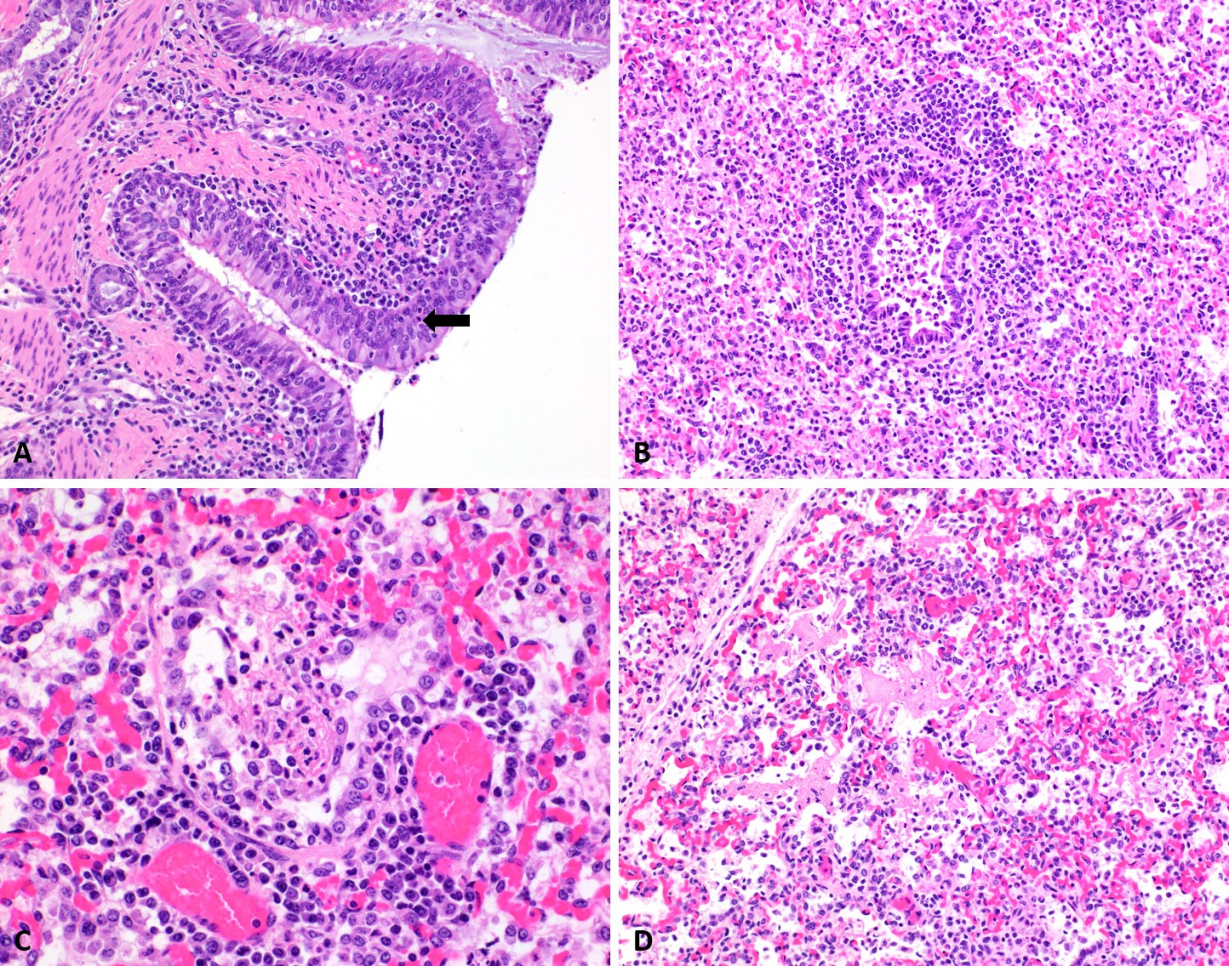
Photomicrographs demonstrating the histological changes encountered. Panel A. The bronchial submucosa is moderately expanded with lymphocytes, plasma cells and occasional macrophages. The periphery of the bronchiole is infiltrated with moderate numbers of lymphocytes, plasma cells and macrophages. The rest of the pulmonary parenchyma is atelectatic, with edema and mixed inflammatory cells within alveolar spaces. Hematoxylin and eosin, 20X. Panel B. In the center, there is a bronchiole containing viable and degenerate neutrophils and a few macrophages. The periphery of the bronchiole is infiltrated with moderate numbers of lymphocytes, plasma cells and macrophages. The rest of the pulmonary parenchyma is atelectatic, with edema and mixed inflammatory cells within alveolar spaces. Hematoxylin and eosin, 20X. Panel C. The bronchiolar lumen contains a fibrin plug with a few entrapped neutrophils and macrophages. This structure is being covered with a low cuboidal to flattened epithelium. This finding was interpreted as early bronchiolitis obliterans. There are small numbers of lymphocytes and plasma cells at the periphery of this bronchiole. Hematoxylin and eosin, 40X. Panel D. Within alveoli, there are moderate numbers of neutrophils and macrophages, together with abundant eosinophilic fibrillary material (fibrin). Alveolar septa are moderately thickened with mononuclear cells. Hematoxylin and eosin, 20X. The S1 File includes additional photomicrographs and the full scoring scheme used.

The unadjusted means and medians are presented in Table 3. The raw data is shown for each individual animal in the matrix plots for the gross pathological and microscopic histological findings (Figs 2–7). The canonical discriminant analyses are presented last and only for microscopic findings (Fig 8). This is followed by the results of the mixed effects regression models. Fig 9 shows examples of some of the major histological findings encountered in examination of the lung sections.

**Table 3:** Mean and median scores for each variable.

	Histopathological score		Viral shedding		Clinical score	
	Mean (SD)	Median (IQR)	Mean (SD)	Median (IQR)	Mean (SD)	Median (IQR)
Ibuprofen	435 (131)	421	1105	54	(130)	68
Day 3		(356–446)	(2298)	(1–827)	(165)	(38–190)
Ibuprofen	656 (317)	421	1105	54	130	68
Day 5		(356–446)	(2298)	(1–827)	(165)	(38–190)
Placebo	603 (436)	417	597	13	155	47
		(255–1094)	(1472)	(0–292)	(146)	(32–186)
FPI	437 (237)	375	459	4	145	84
Day5		(275–625)	(1501)	(0–171)	(143)	(40–176)
FPI + Ibuprofen	538 (315)	510	356	2	106	45
		(276–548)	(826)	(0–63)	(108)	(30–172)
Day 5						
FPI + Ibuprofen Day 3	179 (76)	175	80	0.3	92	54
		(151–191)	(233)	(0–12)	(79)	(36–144)

FPI; fusion protein inhibitor, SD; standard deviation, IQR; interquartile range presented as 25^th^ to 75^th^ centile. Men and median viral shedding and clinical scores were calculated over duration of experiment.

### Consolidation

#### Matrix plots

**Fig 10:**
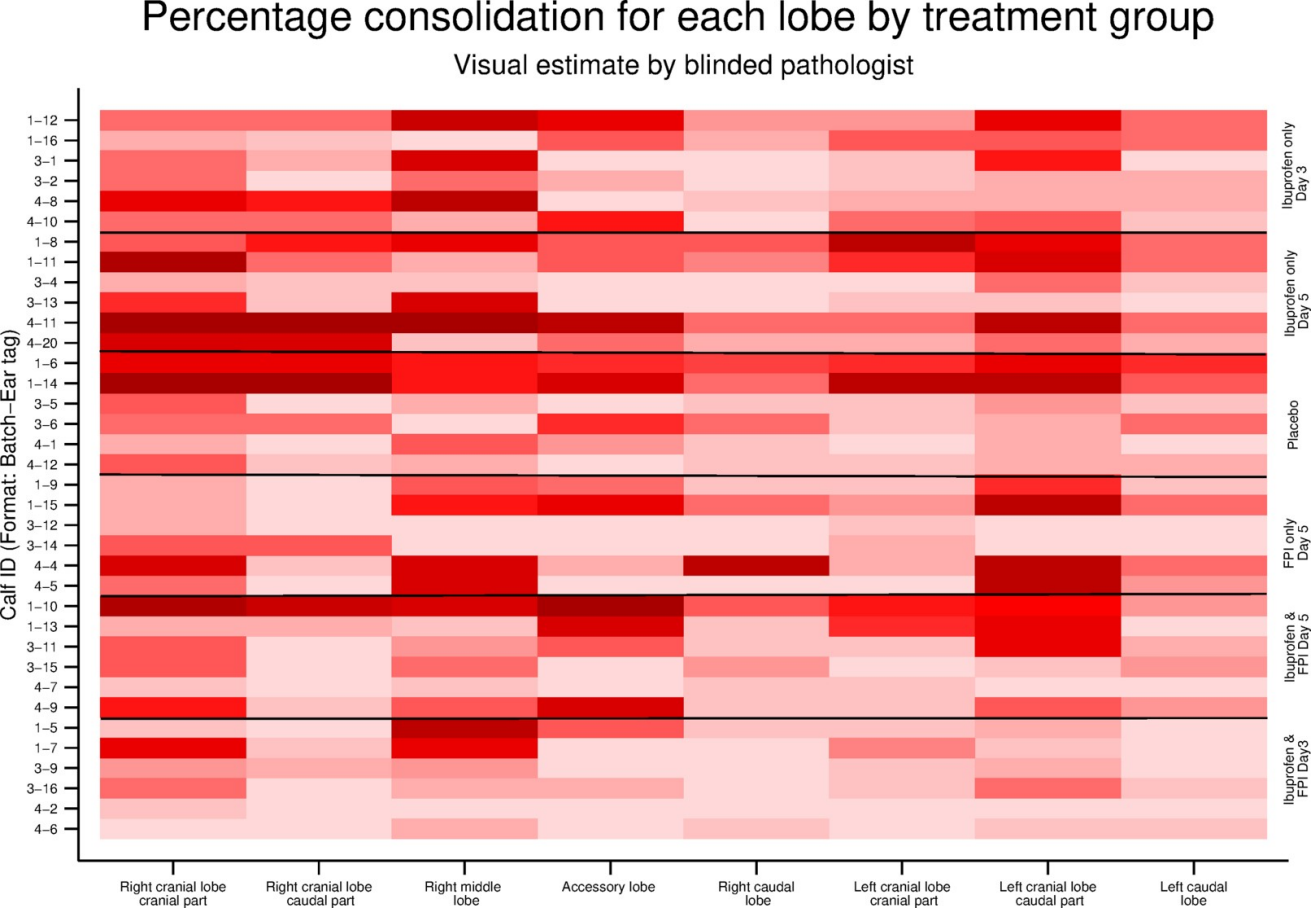
Estimated percentage consolidation for each lobe for each calf by treatment group. Each row represents a single calf. Each column represents a lobe. Darker shading means more and lighter shading less consolidation.

The dependent cranioventral aspect of the lungs (right and left cranial and intermediate loves) tended to be most affected. Inspection of the matrix plot shows a clear benefit in the group that received FPI and ibuprofen from Day 3 (Fig 10). The differences between the other treatment groups are less apparent on simple visual inspection although FPI treated groups did appear to have less consolidation. The last four columns represent quartiles of mean peak and clinical scores and mean and peak viral loads measured by nasal swabs and are therefore the same for each graph.

#### Mixed effects regression

Compared to placebo, consolidation was statistically significantly decreased only in those calves receiving both ibuprofen and FPI on day 3 following inoculation (*p* = 0.001) (Table 3). *Mycoplasma bovis* was prevalent in the first replicate but not the others. The isolated bacteria are detailed in (S2 File). Consolidation was significantly different in the multilevel regression only for the group who received both ibuprofen and FPI on day 3 when compared to placebo (n = 6 β = -151, *p* = 0.015) (Table 3). Pairwise comparison of the results for each treatment group are shown in Table 5. We did not see increased consolidation when ibuprofen was started on day 5 rather than day 3, or when FPI or ibuprofen monotherapy was started on day 5. Otherwise, the results for consolidation broadly parallel those for the histology but are less pronounced (discussed below).

### Histology

#### Matrix plots

Inspection of the matrix plots (darker squares meaning more severe) shows that the cranial dependent lobes of the lung, and particularly the right lung, consistently had the greatest histological changes in most structures regardless of treatment group. The last four cells for each animal show the quartile for mean and peak daily clinical scores and mean and peak viral shedding for that animal are therefore the same for each graph.

Animals in the first batch/replicate tended to have more severe histological changes, clinical scores, and more viral shedding than the other replicates. Animals in the placebo group had the worst clinical scores, those in the ibuprofen only groups had the highest viral shedding.

Calves who received FPI and ibuprofen from Day 3 had the best results. Histological differences between groups at day 10 post inoculation were most apparent at the levels of the pleura, (Fig 2) alveolus (Fig 3). The differences between treatment groups were less obvious in the histology of the bronchioles (Fig 4), bronchus (Fig 5), and septum (Fig 6). There were no discernable differences in histology outcomes at the level of the interstitium (Fig 7).

#### Mixed effects regression of microscopic findings

In the combined multilevel analysis only the treatment group receiving ibuprofen and FPI three days after inoculation reached statistical significance with respect to histological changes compared to placebo (n = 6 β = -122, *p* <0.001) FPI alone at 5 days (n = 6, β -46, *p* = 0.089) had a modest non-significant benefit, albeit with small *N*. Pairwise comparison of the results for each treatment group are shown in Table 4.

**Table 4:** A. Results for multilevel models for the overall pathology score. B. Results for multilevel models for the histological and consolidation components of the overall pathology score.

A.														
		Overall Pathology Score												
		Change in score		p value			95%Cl							
							lb		Ub					
Ibuprofen only Day 3		-162		0.208			-415		91					
Ibuprofen only Day 5		58		0.652			-195		310					
FPI only Day 5		-162		0.207			-414		90					
Ibuprofen and FPI Day 5		-69		0.589			-321		182					
Ibuprofen and FPI Day 3		**-425**		**0.001**			**-676**		**-173**					
B.														
	Histology							Consolidation						
	Change in score		p value		95%Cl			Change in score		p value			95%Cl	
					lb	ub							lb	ub
Ibuprofen only Day 3	-38		0.161		-415	91		-63			0.315		-185	59
Ibuprofen only Day 5	-19		0.497		-72	35		38			0.544		-84	160
FPI only Day 5	-46		0.089		-100	7		-58			0.348		-18	63
Ibuprofen & FPI Day 5	-32		0.247		-85	22		-19			0.763		-140	103
Ibuprofen & FPI Day 3	-122		**<0.001**		**-175**	**-69**		**-151**			**0.015**		**-273**	**-30**

Placebo is referent. Cl; confidence interval, lb; lower bound, ub upper bound.

There are some sharp contrasts between the histological and clinical outcomes. Whereas clinical outcomes were virtually identical for those calves started on either ibuprofen alone or FPI alone on day 5 following infection there were distinct histological differences (n = 6, β+214 p+0.042) favoring FPI. This difference favoring FPI persists even when the clinical score is calculated without temperature.

Differences in viral shedding also seems inadequate to explain our findings. Starting ibuprofen on day 5 post infection caused more severe histological injury than starting ibuprofen on day 3 despite viral loads being higher in those started on day 3 (mean 1105, median 54 viral copies in the ibuprofen starting day 3 group, versus 701 and 11 viral copies in the ibuprofen starting day 5 group). The implication is that neither histological changes as determined by H&E staining nor viral shedding fully explain the observed clinical severity of bovine RSV infection.

#### Canonical discriminant analysis

Fig 8 shows the score plots for the canonical discriminant analysis. These findings reflect both the timing of necropsy, after peak clinical illness when recovery had started, the sites of maximal injury caused by RSV, and the locations of benefit of each drug. Separation was greatest at the level of the alveolus, septum, and pleura. There was less separation at the level of the bronchiole and bronchus. The separation of all six groups does underscore that early rather than late timing of drug initiation does lead to different histological outcomes. Given that ‘late” was only 5 days following infection these graphs further highlight the need for early diagnosis. Not all tissue demonstrated evidence of a drug effect. Overlap rather than separation occurred between treatment groups at the level of the interstitium (Fig 8).

#### Mixed effects regression of combined histopathological score

In multilevel regression only the treatment group receiving dual therapy with ibuprofen and FPI three days after infection reached statistical significance with respect to overall histopathological score when compared with placebo (Table 4A). FPI alone at 5 days and ibuprofen alone at 3 days following infection had a similar albeit not statistically significant effects on the histopathological score. The likelihood ratio test favored multilevel regression over ordinary least squares linear regression (x^2^ = 8.60, p = 0.0017)

The results of pairwise comparison of the different treatment groups and the results that would have been obtained if only those two groups had been included in the experiment are shown in Table 5A.

**Table 5:** A. Pairwise comparison of the overall treatment score and histology component for each pair of treatments analyzed separately (as if we had performed an individual experiment with two groups of six animals), and together. B. Pairwise comparison of the consolidation component of the overall treatment score and the virus copies for each pair of treatments analyzed separately (as if we had performed an individual experiment with two groups of six animals), and together.

A.								
Treatment group	Individual analysis for each treatment pair		All groups analyzed together		Individual analysis for each treatment pair		All groups analyzed together	
	Total Score		Total Score		Histology score		Histology score	
	Chane in score	p	Change in score	p	Change in score	p	Change in score	p
Ibu Day 5 vs. Ibu Day 3	**219**	**0.026**	**220**	**0.086**	100	0.037	100	0.105
FPI & Ibu Day 3 vs.Ibu Day 3	**-274**	**<0.001**	**-263**	**0.042**	-100	<0.001	-89	0.155
FPI Day 5 vs. Ibu Day 5	-214	0.042	-220	0.086	-94	0.074	-96	0.121
FPI & Ibu Day 3 vs.Ibu Day 5	**-502**	**<0.001**	**-483**	**<0.001**	-203	<0.001	-189	0.002
FPI & Ibu Day 3 vs. Placebo	**-425**	**0.002**	**-425**	**0.001**	-151	0.017	-151	0.015
FPI & Ibu Day 3 vs.FPI Day 5	**-237**	**0.011**	**-263**	**0.041**	-83	0.046	-83	0.134
FPI & Ibu Day 3 vs. FPI & Ibu Day 5	-359	0.003	-356	0.006	-134	0.015	-133	0.032
B.								
Treatment group	Individual analysis for each treatment pair		All groups analyzed together		Individual analysis for each treatment pair		All groups analyzed together	
	Consolidation score		Consolidation score		Viral copies		Viral copies	
	Change in score	p	Change in score	p	Change in viral load	P	Change in viral load	P
Ibu Day 5 vs. Ibu Day 3	19	0.168	20	0.470	-359	0.318	-362	0.127
FPI & Ibu Day 3 vs.Ibu Day 3	-75	<0.001	-84	0.002	-992	<0.001	-979	<0.001
FPI Day 5 vs. Ibu Day 5	-25	0.192	-28	0.308	-241	0.367	-242	0.302
FPI & Ibu Day 3 vs. Ibu Day5	-95	<0.001	-104	<0.001	-614	0.016	-616	0.008
FPI & Ibu Day 3 vs. Placebo	-123	<0.001	-123	<0.001	-992	<0.001	-469	0.049
FPI & Ibu Day 3 vs. FPI Day 5	-71	<0.001	-76	0.005	-375	0.037	-375	0.108
FPI & Ibu Day 3 vs. FPI & Ibu Day 5	-91	<0.001	-91	0.001	-285	0.002	-296	0.205

Negative scores are better. Comparison is reported as the difference in score if the first rather than second drug in the treatment group was administered. The first two columns for each outcome report the coefficients and the p-values for each comparison. These models included only the 12 animals in the direct comparison. The individual pairwise comparisons are not adjusted for multiple comparisons. The second two columns report the results from the same single main model which included all the data. FPI; fusion protein inhibitor. Ibu: ibuprofen, p: p-value. The unit is score.

Pairwise comparison (Table 5A) emphasizes how the addition of FPI to ibuprofen led to lower histopathological scores. FPI may lead to less lung damage than ibuprofen alone (score lower by 214 points (p = 0.086) than does ibuprofen at 5 days). Adding FPI to ibuprofen starting on day 3 caused the histopathological score to be 274 (p = 0.042) points lower than using ibuprofen alone over the same period. These comparisons also underline the importance of starting treatment early. The optimal treatment combination of ibuprofen and FPI dual therapy started on day 3 led to lower scores (359 p = 0.006) compared to treatment started on day 5. Finally, there may be potential for ibuprofen monotherapy to cause harm if initiated later; ibuprofen started on day 5 led to higher histopathological scores than did ibuprofen started on day 3 (p = 0.086).

## Discussion

We have used a three-pronged approach to the inherent difficulty in reporting histopathology results where the number of possible combinations of histological findings far exceeds the number of animals that any randomized controlled trial could reasonably include. First, we used matrix plots, labeled to the detail of each structure; each slide; each lung lobe; for each animal, to present all of the data captured by the pathologist allowing the reader to visually estimate the differences between treatment groups. Matrix plots do not summarize the data, quite the contrary. The alternative matrix plots would be a table. In such a table each colored square from the matrix plot would be replaced by a number. Such a table would be impenetrable. Second, we used histopathological scoring system which we have previously shown to correlate with clinical status in life. We then used these scores as the dependent variables in mixed effect regressions. Mixed effects, also called multilevel, or hierarchical regressions, model the data generating process. This is particularly important when doing resource intensive experiments in batches. It also is a correct method of analysis following the use of data minimization, a type of randomization that improves statistical power. The statistical code required to allow others use this approach is provided in (S3 File). Third, we used discriminant analysis as a variable reduction technique to visualize the pathological differences between treatment groups. Canonical discriminant analysis emphasizes difference rather than similarity. Canonical discriminant analysis does exclude colinear and uninformative variables, but the information it loses is retained in the matrix plots and mixed effects regressions. Unlike the way in which the scoring system reduced all the variables (even uninformative and colinear ones) to a single score, canonical discriminant analysis is a multivariant method; its functions are linear combinations of variables that best separate the mean vectors of two or more groups of multivariate observations relative to the within-group variance [39]. The statistical code required to facilitate others to use this approach is in the (S4 File). We believe that presenting the results of these three analytical methods and the full scoring scheme used along with representative photomicrographs provides a reproducible and comprehensive report of our findings that may be more accessible to non-pathologists than reporting photomicrographs and scores alone.

We found beneficial changes in lung histology in animals treated with both ibuprofen and FPI at 3 days following inoculation. This effect is readily apparent from the matrix plots and was statistically significant in regression analysis despite the small sample size. Canonical discriminant analysis demonstrated separation between the other treatment groups that is less apparent on simple inspection of the raw data. In the case of interstitial histology canonical discriminant analysis did not find separation between treatment groups.

Except for those animals receiving ibuprofen alone from day 5, we found that histological changes, amount of consolidation, and overall histopathology score were lower in every treatment group than placebo. This difference between treatments and placebo was statistically significant in those animals receiving FPI and ibuprofen from day 3.

Our analyses showed similarities and contrasts with the clinical outcomes study. The best was treatment was dual therapy with ‘ibuprofen and FPI started on Day 3’ in both clinical ant pathological studies. Inspection of the matrix plots and regression coefficients suggests ‘FPI alone started on Day 5’ to be the second most beneficial treatment. This contrasts our study of clinical outcomes which showed ‘FPI and ibuprofen started on Day 5’ to be the second most beneficial treatment and this was statistically significant [16].

There are some possible explanations for this. First, the clinical study (modestly) gained statistical power from the repeated measurements of clinical outcomes performed over the course of the illness, whereas necropsy is a single event. Second, clinical outcomes are influenced by fever and hydration. A calf may be doing relatively better clinically because fever control leads to increased oral intake which in turn leads to better hydration, activity, clinical scores, and weight gain even though FPI rather than ibuprofen might have led to better histological outcomes. Third, other factors responsive to drug treatment that are not detectable with H&E staining such as neutrophil extracellular traps (NET) may influence outcomes.

Our rationale for including ibuprofen is its potential role as an immunomodulator [40–43]. We have previously shown that ibuprofen started a day following inoculation decreases the range of Il-17 and Il-13 production with respect to interferon γ and IL-4 [12]. This did not translate to a change in histology analyzed when started on day 1, 3 or 5 post inoculation. One potential explanation may be that ibuprofen is affecting the activity of NETs. These are extracellular DNA fibers covered with histones, and cytotoxic proteins, such as elastase and myeloperoxidase that capture and inactivate bacteria, fungi, and some viruses including RSV [44, 45]. These traps contribute to mucous plugging and in the case of RSV may do so excessively; a third of obstructing NET containing mucous plugs do not contain virus [46, 47]. We are currently investigating this possibility.

Alternative possibilities such as bronchospasm would not be expected to be relieved by NSAIDS, FPI, or our placebo preparations.

The current experiment found worse results when ibuprofen was started on day 5 rather than day 3, although this was not statistically significant when all groups were tested simultaneously (*p* = 0.026 direct versus *p* = 0.086 group comparison). NSAIDs and FPI started singly on day 5 post inoculation showed similar clinical benefit [16]. The histopathology for each of these groups failed to show a significant difference (β = -220 *p* = 0.089 in pairwise comparison,β = -214 *p* = 0.042 unadjusted). This raises the possibility that just as early diagnosis is essential for FPI to be of benefit, early diagnosis might also be necessary to prevent ibuprofen doing harm. It also suggests that despite ibuprofen improving clinical scores there may be a cost in terms of histological outcomes. When, as is inevitable in clinical practice, time of infection is uncertain FPI alone is preferred to ibuprofen with respect to histological outcomes if monotherapy with one drug is the only option. The more immediately plausible clinical dilemma (because ibuprofen is inexpensive and FPIs are generally unavailable or expensive) is when faced with a sick patient deciding if it is already too late to start treatment. This will require strategies geared to early diagnosis and the cost benefit ratio of these will depend on the species, and cose and side effects of the drugs used

### Transferability

Our work applies directly to cattle in general and Holsteins in particular. Implementing these results clinically would require a commercial a pricing decision by pharmaceutical companies to sell FPI. Because of the potential for resistance a two-antiviral drug approach would seem advisable. The bovine model of respiratory syncytial virus is readily transferable to humans [48, 49]. The NSAID we chose, ibuprofen, is more commonly used in pediatrics than agriculture where longer acting agents are preferred. The FPI (GS-561937) we used differs from its experimental human counterpart drug (GS-5806) only in the substitution of a methyl group for a Cl in the bovine version [17, 18]. This transferability is useful because histology is almost never available in human infants. Our work further underscores the necessity for early diagnosis if dual antiviral-NSAID therapy is to be effective. While clinical scoring systems show a benefit of treatment up to five days following inoculation, achieving even earlier diagnosis offers the promise of decreased lung injury on histology.

We speculate that this need for early diagnosis would also hold true for human infants. For pediatrics this suggests a degree of clinical nuance will be needed to balance the improvement in clinical scores [12] and perhaps decreased risk of later wheezing [14] achieved by modulation the immune response with an NSAID against not merely increased viral shedding [12] but potentially also increased consolidation and microscopic damage is ibuprofen is started late. Those practitioners using lung ultrasound may be able to immediately leverage our findings with respect to NSAIDs in both veterinary and pediatric emergency medicine.

### Limitations

The primary limitation is small sample size. Our work does beg the question: How would an FPI only on Day 3 group have fared in comparison to the dual therapy? Jordan *et al* compared placebo with intravenous rather than oral GS-5806 at Days 1 and 3 following experimental BRSV infection. There was less consolidation and less histological injury in the group treated at 3 days post inoculation than placebo [18]. Direct comparison between these studies is difficult but may be feasible in a network meta-analysis. Other limitations include the inherent variability in outbred animals, the use of a single rather than multiple overlapping pathologists to interpret the slides and not having that pathologist over read his own slides to calculate intrareader reliability.

In conclusion, we found that adding FPI to NSAIDs leads to less lung injury than either agent alone. The clinical benefits of combined antiviral and NSAID treatment of bovine RSV extend at least partially from histopathological changes in the lung when treatment was started three days after infection, but we failed to show that the clinical benefits seen at five days post infection can be explained by solely histological differences, as identified using H&E staining, in outcome particularly when treatment is started five days after infection.

## Supporting information

S1 File (PDF)

S2 File (PDF)

S3 File Statistical code for matrix plots. (DOC)

S4 File Statistical code needed to perform canonical discriminant analysis. (DOC)

S5 File (DTA)
