# Supplementary material for: A randomized controlled trial comparing non-steroidal anti-inflammatory and fusion protein inhibitors singly and in combination on the histopathology of bovine respiratory syncytial virus infection
Source: PLoS One. 2021 Jun 10;16(6):e0252455. doi: 10.1371/journal.pone.0252455 (PMC8191941; doi:10.1371/journal.pone.0252455)
Supplement: S1 File — (PDF) [file pone.0252455.s001.pdf]

Histologic grading of pulmonary lesions

Pathologist: Francisco R Carvallo DVM, DSc, DACVP Analyst: Paul Walsh MB BCh

April 17, 2021

Contents

1 Description of scoring schema **5**

1.1 Bronchus . . . 5
1.1.1 Neutrophilic exudate . . . 5
1.1.2 Deciliation . . . 5
1.1.3 Epithelial transmigration . . . 5
1.1.4 Intraepithelial pustules . . . 6
1.1.5 Inclusion bodies . . . 6
1.1.6 Bronchial mononuclear infiltrates . . . 6
1.1.7 Lymphoid nodules . . . 6

1.2 Bronchioles . . . 7
1.2.1 Neutrophilic exudate . . . 7
1.2.2 Fibrinous exudate . . . 7
1.2.3 Bronchiolitis obliterans . . . 7
1.2.4 Necrosis of bronchiolar epithelium . . . 7
1.2.5 Inclusion bodies . . . 8
1.2.6 Epithelial transmigration . . . 8
1.2.7 Peribronchiolar mononuclear infiltrates . . . 8
1.2.8 Peribronchiolar lymph nodes . . . 8

1.3 Alveolus . . . 8
1.3.1 Neutrophilic exudate . . . 9
1.3.2 Fibrinous exudate . . . 9
1.3.3 Necrosis . . . 9
1.3.4 Edema . . . 9
1.3.5 Hemorrhages . . . 9
1.3.6 Syncytial cells . . . 10
1.3.7 Type II pneumocyte hyperplasia . . . 10
1.3.8 Granulomas . . . 10
1.3.9 Atelectasis . . . 10
1.3.10 Lytic necrosis . . . 10

1.4 Interstitium . . . 11
1.4.1 Thickening with mononuclear cells . . . 11
1.4.2 Thickening with polymorphonuclear cells . . . 11
1.4.3 Vasculitis . . . 11
1.4.4 Thrombosis . . . 11

1.4.5 Lymphoid nodules . . . 12
1.5 Septum . . . 12
1.5.1 Expansion . . . 12
1.5.2 Edema . . . 12
1.5.3 Fibrin . . . 12
1.5.4 Mononuclear cell infiltrate . . . 13
1.5.5 Neutrophil infiltrates . . . 13
1.5.6 Pleocellular infiltrates . . . 13
1.5.7 Vasculitis . . . 13
1.5.8 Fibrosis . . . 13
1.6 Pleura . . . 14
1.6.1 Thickening . . . 14
1.6.2 Fibrosis . . . 14
1.6.3 Lymphatic dilation/edema . . . 14
1.6.4 Mononuclear cell infiltrate . . . 14
1.6.5 Polymorphonuclear infiltrates . . . 15
1.6.6 Pleocellular infiltrates . . . 15

2 Sample Photomicrographs 17

3 Data Entry 25

4 Plotting the results using Matrix plots 27
4.1 Statistical code for Matrix plots . . . 27

5 Performing Canonical Discrimination 29
5.1 Statistical code needed to perform canonical discriminant analysis 29
5.1.1 Improving the graphs . . . 30

Chapter 1

Description of scoring schema

1.1 Bronchus

Score each slide as 0 to 3 for each finding

1.1.1 Neutrophilic exudate

Neutrophilic exudate: Presence of neutrophils in the lumen of bronchi.

0: No neutrophils in bronchi.
1: Occasional neutrophils in bronchi.
2: Moderate numbers of neutrophil in bronchi, without occlusion of the lumen.
3: numerous neutrophils in bronchi, with more than 80% occlusion of the lumen.

1.1.2 Deciliation

Deciliation: Characterized by the absence of cilia at the apical margin of bronchial epithelial cells.

0: No deciliation.
1: Occasional deciliation of epithelial cells.
2: Less than 50% of deciliated epithelial cells.
3: More than 50% deciliated epithelial cells.

1.1.3 Epithelial transmigration

Epithelial transmigration: Characterized by the presence of intrabronchial epithelial neutrophils.

0: No transmigration of neutrophils.
1: Occasional intraepithelial neutrophils.
2: Moderate numbers (less than 10) of intraepithelial neutrophils.
3: Numerous (more than 10) intraepithelial neutrophils.

1.1.4 Intraepithelial pustules

Intraepithelial pustules: characterized by the presence of clusters of neutrophils (more than three neutrophils per cluster) in the mucosa of a bronchus.

0: No intraepithelial pustules.
1: One or two intraepithelial pustules.
2: Three of four intraepithelial pustules.
3: More than five intraepithelial pustules.

1.1.5 Inclusion bodies

Inclusion bodies: Presence of eosinophilic inclusions in the cytoplasm of epithelial cells.

0: No inclusion bodies.
1: Inclusion bodies in one bronchus.
2: Inclusion bodies in two bronchi.
3: Inclusion bodies in three or more bronchi.

1.1.6 Bronchial mononuclear infiltrates

Bronchial mononuclear infiltrates: Characterized by the presence of lymphocytes, plasma cells and/or macrophages in the bronchial wall.

0: No mononuclear infiltrates.
1: Small numbers of mononuclear cells, above the muscularis mucosa.
2: Small numbers of mononuclear cells above and below the muscularis mucosa.
3: Large numbers of mononuclear cells above and below the muscularis mucosa.

1.1.7 Lymphoid nodules

Lymphoid nodules: Presence of nodular lymphoid aggregates at the periphery of bronchi.

0: No peri bronchial lymphoid nodules.
1: Occasional peri bronchial lymphoid nodules (less than 2 per section).
2: Between 3-5 peri bronchial lymphoid nodules.
3: More than 6 peri bronchial lymphoid nodules.

1.2 Bronchioles

Score each slide as 0 to 3 for each finding

1.2.1 Neutrophilic exudate

Neutrophilic exudate: Presence of neutrophils in the lumen of bronchioles.

0: No neutrophils in bronchioles.
1: Occasional neutrophils in a few bronchioles.
2: Occasional neutrophils in multiple bronchioles (less than half in the section)
3: numerous neutrophils on most bronchioles.

1.2.2 Fibrinous exudate

Fibrinous exudate: Presence of fibrin in the lumen of bronchioles.

0: No fibrin in bronchioles.
1: Small amounts of fibrin in occasional bronchioles.
2: Small amounts of fibrin in multiple bronchioles (less than half in the section).
3. Abundant fibrin in most bronchioles.

1.2.3 Bronchiolitis obliterans

Bronchiolitis obliterans: Presence of polyp-like projections of the mucosa that partially or totally occlude the bronchiolar lumen.

0: No bronchiolar polyp-like structure in the section.
1: One bronchiolar polyp-like structure in the section.
2: Two bronchiolar polyp-like structure in the section.
3: Three or more bronchiolar polyp-like structures in the section.

1.2.4 Necrosis of bronchiolar epithelium

Necrosis of bronchiolar epithelium: Characterized by the presence of cells with hypereosinophilic cytoplasm and pyknotic/karyorrhectic nucleus in bronchioles.

0: No necrotic cells in bronchioles
1: Occasional necrotic cells in few bronchioles.
2: Occasional necrotic cells in multiple bronchioles.
3: Numerous necrotic cells in multiple bronchioles.

1.2.5 Inclusion bodies

Inclusion bodies: Presence of eosinophilic inclusions in the cytoplasm of epithelial cells.

0: No inclusion bodies.
1: Inclusion bodies in one bronchiole.
2: Inclusion bodies in two bronchioles.
3: Inclusion bodies in three or more bronchioles.

1.2.6 Epithelial transmigration

Epithelial transmigration: Characterized by the presence of neutrophils in the mucosa of bronchioles.

0: No transmigration of neutrophils.
1: Occasional intraepithelial neutrophils in a few bronchioles.
2: Occasional intraepithelial neutrophils in multiple bronchioles.
3: Numerous intraepithelial neutrophils in multiple bronchioles.

1.2.7 Peribronchiolar mononuclear infiltrates

Peribronchiolar mononuclear infiltrates: Characterized by the presence of lymphocytes, plasma cells and/or macrophages in the propria submucosa.

0: No peribronchiolar mononuclear infiltrates.
1: Small numbers of mononuclear cells in a few bronchioles.
2: Small numbers of mononuclear cells in multiple bronchioles.
3: Large numbers of mononuclear cells in multiple bronchioles.

1.2.8 Peribronchiolar lymph nodes

Peribronchiolar lymph nodes: Presence of nodular lymphoid aggregates at the periphery of bronchioles.

0: No peribronchiolar lymph nodes.
1: Occasional peribronchiolar lymph nodes (less than 2 per section).
2: Between 3-5 peribronchiolar lymph nodes.
3: More than 6 peribronchiolar lymph nodes.

1.3 Alveolus

Score each slide as 0 to 3 for each finding

1.3.1 Neutrophilic exudate

Neutrophilic exudate: **

0: No neutrophils in alveolar spaces.
1: Occasional neutrophils in alveolar spaces.
2: Accumulation of neutrophils in alveolar spaces, without occluding the lumen.
3: Occlusion of the lumen with large numbers of neutrophils.

1.3.2 Fibrinous exudate

Fibrinous exudate: Characterized by the presence of a fibrillary eosinophilic material.

0: No fibrin in alveolar spaces.
1: Small amounts of fibrin in occasional alveolar spaces.
2: Small amounts of fibrin in multiple alveolar spaces.
3. Abundant fibrin in multiple alveolar spaces.

1.3.3 Necrosis

Necrosis: Characterized by the presence of cells with hypereosinophilic cytoplasm and pyknotic/karyorrhectic nucleus in alveolar spaces.

0: No necrotic cells in alveolar spaces
1: Occasional necrotic cells in few alveolar spaces.
2: Occasional necrotic cells in multiple alveolar spaces.
3: Numerous necrotic cells in multiple alveolar spaces.

1.3.4 Edema

Edema: Characterized by the presence of an amorphous eosinophilic material.

0: No edema.
1: Small amounts of edema in occasional alveolar spaces.
2: Small amounts of edema in multiple alveolar spaces.
3. Abundant edema in multiple alveolar spaces.

1.3.5 Hemorrhages

Hemorrhages: Characterized by the presence of extravasated erythrocytes.

0: No hemorrhages.
1: A few erythrocytes in occasional alveolar spaces.
2: A few erythrocytes in multiple alveolar spaces.
3. Numerous erythrocytes in multiple alveolar spaces.

1.3.6 Syncytial cells

Syncytial cells: Characterized by the presence of multinucleated cells in the lumen of alveolar spaces, not associated with the presence of fibrin.

0: No syncytial cells.
1: Occasional syncytial cells in a few alveolar spaces.
2: Occasional syncytial cells in multiple alveolar spaces.
3: Numerous syncytial cells in multiple alveolar spaces.

1.3.7 Type II pneumocyte hyperplasia

Type II pneumocyte hyperplasia: Characterized by the presence of cuboidal cells covering the alveolar surface.

0: No type II pneumocyte hyperplasia.
1: Segmental hyperplasia of type II pneumocytes in a few alveolar spaces.
2: Segmental hyperplasia of type II pneumocytes in multiple alveolar spaces.
3: Diffuse hyperplasia of type II pneumocytes.

1.3.8 Granulomas

Granulomas: Characterized by the presence of a core of necrosis containing necrotic cellular debris and neutrophils, all surrounded with macrophages, lymphocytes and plasma cells and a peripheral capsule of fibrous connective tissue.

0: No granulomas.
1: One granuloma in the section.
2: Two granulomas in the section.
3: Three or more granulomas in the section.

1.3.9 Atelectasis

Atelectasis: characterized by alveolar collapse.

0: No atelectasis.
1: Focal area(s) of atelectasis, overall comprising less than 20% of the section.
2: Focal area(s) of atelectasis, overall comprising between 20-50% of the section.
3: Focal areas of atelectasis, comprising more than 50% of the section or diffuse atelectasis.

1.3.10 Lytic necrosis

Lytic necrosis: Characterized by the presence of well demarcated areas of lytic (liquefactive) necrosis in alveolar spaces/septa.

0: No areas of liquefactive necrosis.
1: One area of liquefactive necrosis.
2: Two areas of liquefactive necrosis.
3: Three or more areas of liquefactive necrosis.

1.4 Interstitium

Score each slide as 0 to 3 for each finding

1.4.1 Thickening with mononuclear cells

Thickening with mononuclear cells: presence of lymphocytes, plasma cells and/or macrophages within the alveolar wall.

0: No thickening (compared to control)
1: Mild thickening (small numbers of mononuclear cells).
2: Moderate thickening (Moderate numbers of mononuclear cells).
3: Severe thickening

1.4.2 Thickening with polymorphonuclear cells

Thickening with polymorphonuclear cells: Presence of neutrophils or eosinophils within the alveolar wall.

0: No polymorphonuclear cells.
1: focal small infiltrate of polymorphonuclear cells.
2: Multifocal small infiltrates of polymorphonuclear cells.
3: Multifocal to diffuse infiltrates of polymorphonuclear cells.

1.4.3 Vasculitis

Vasculitis: Presence of inflammatory cells in the vascular wall.

0: No vasculitis.
1: Inflammatory cells in the wall of one vessel.
2: Inflammatory cells in the wall of two vessels.
3: Inflammatory cells in the wall of three or more vessels.

1.4.4 Thrombosis

Thrombosis: Presence of a fibrin plug partially or totally occluding the lumen of an alveolar capillary.

0: No thrombus.
1: Occasional thrombi scattered in the section.
2: Thrombi in multiple capillaries.
3: At least one thrombus in each high magnification power field (40X).

1.4.5 Lymphoid nodules

Lymphoid nodules: Presence of nodular lymphoid aggregates in the interstitium.

0: No interstitial lymphoid nodules.
1: Occasional interstitial lymph nodes (less than 2 per section).
2: Between 3-5 interstitial lymphoid nodules.
3: More than 6 interstitial lymphoid nodules.

1.5 Septum

Score each slide as 0 to 3 for each finding

1.5.1 Expansion

Expansion: General observation of the interlobular septa based on the presence of fibrin, edema and/or inflammatory cells)

0: No expansion (compared to the control)
1: Mild expansion (double the amounts of fibrin and/or edema and/or inflammatory cells) compared to 0.
2: Moderate expansion (Triple the amounts of fibrin and/or edema and/or inflammatory cells) compared to 0.
3: Severe expansion (more than 4 times of fibrin and/or edema and/or inflammatory cells) compared to 0.

1.5.2 Edema

Edema: Characterized by the presence of transparent spaces between fibers of collagen.

0: No lymphatic dilation/edema (control)
1: Mild lymphatic dilation/edema
2: Moderate lymphatic dilation/edema
3: Severe lymphatic dilation/edema

1.5.3 Fibrin

Fibrin: Presence of a fibrillary eosinophilic material in the interlobular septum.

0: No fibrin in interlobular septa.
1: Small amounts of fibrin in occasional septa.
2: Small amounts of fibrin in multiple septa.
3. Abundant fibrin in multiple septa.

1.5.4 Mononuclear cell infiltrate

Mononuclear (lymphocytes, plasma cells and/or macrophages) cell infiltrate:

0: No mononuclear cells (control)
1: Mild mononuclear infiltrates (a few scattered cells)
2: Moderate mononuclear infiltrate (between 10-30 mononuclear cells per high magnification field (40x))
3: Severe mononuclear infiltrate (More than 30 cells per high magnification field)

1.5.5 Neutrophil infiltrates

Neutrophil (polymorphonuclear) infiltrates:

0: No neutrophils (control)
1: Mild neutrophilic infiltrates (A few scattered cells))
2: Moderate neutrophilic infiltrate (between 10-30 mononuclear cells per high magnification field (40x))
3: Severe neutrophilic infiltrate (More than 30 cells per high magnification field)

1.5.6 Pleocellular infiltrates

Pleocellular (polymorphonuclear and mononuclear mixed) infiltrates

0: No pleocellular infiltrates (control)
1: Mild pleocellular infiltrates (A few scattered cells))
2: Moderate pleocellular infiltrate (between 10-30 cells per high magnification field (40x))
3: Severe pleocellular infiltrate (More than 30 cells per high magnification field)

1.5.7 Vasculitis

Vasculitis: Presence of inflammatory cells within the vascular wall.

0: No vasculitis.
1: Inflammatory cells in the wall of one vessel.
2: Inflammatory ells in the wall of two vessels.
3: Inflammatory cells in the wall of three or more vessels.

1.5.8 Fibrosis

Fibrosis: Expansion of the septum with collagen.

0: same amounts of connective tissue (compared to the control)

1: Mild fibrosis (double the amounts of connective tissue compared to control)
2: Moderate fibrosis (Triple the amounts of connective tissue compared to control)
3: Severe fibrosis (More than 4 times the amount of connective tissue compared to control).

1.6 Pleura

1.6.1 Thickening

Thickening: Sub-gross (4X) observation of the pleura based on the presence of connective tissue (fibrosis), edema and/or inflammatory cells)

0: No thickening (compared to the control)
1: Mild thickening (double the amounts of connective tissue and/or edema and/or inflammatory cells) compared to 0.
2: Moderate thickening (Triple the amounts of connective tissue and/or edema and/or inflammatory cells) compared to 0.
3: Severe thickening (more than 4 times of connective tissue and/or edema and/or inflammatory cells) compared to 0.

1.6.2 Fibrosis

Fibrosis: characterized by the presence of collagen in the pleura.

0: same amounts of collagen (compared to the control)
1: Mild fibrosis (double the amounts of collagen compared to control)
2: Moderate fibrosis (Triple the amounts of collagen compared to control)
4: Severe fibrosis (More than 4 times the amount of collagen compared to control).

1.6.3 Lymphatic dilation/edema

Lymphatic dilation/edema: Lymphatic dilation is characterized by the presence of ectatic lymph vessels. Edema is characterized by the presence of an extracellular eosinophilic proteinaceous amorphous material.

0: No lymphatic dilation/edema (control)
1: Mild lymphatic dilation/edema
2: Moderate lymphatic dilation/edema
3: Severe lymphatic dilation/edema

1.6.4 Mononuclear cell infiltrate

Mononuclear cell infiltrate: Characterized by the presence of lymphocytes, plasma cells and/or macrophages

0: No mononuclear cells (control)
1: Mild mononuclear infiltrates (a few scattered cells)
2: Moderate mononuclear infiltrate (between 10-30 mononuclear cells per high magnification field (40x))
3: Severe mononuclear infiltrate (More than 30 cells per high magnification field)

1.6.5 Polymorphonuclear infiltrates

Polymorphonuclear(PMNs) infiltrates: Characterized by the presence of neutrophils and/or eosinophils.

0: No PMNs (control)
1: Mild PMNs infiltrates (A few scattered cells))
2: Moderate PMNs infiltrate (between 10-30 mononuclear cells per high magnification field (40x))
3: Severe PMNs infiltrate (More than 30 cells per high magnification field)

1.6.6 Pleocellular infiltrates

Pleocellular infiltrates: Characterized by the presence of polymorphonuclear and mononuclear mixed inflammatory infiltrates.

0: No pleocellular infiltrates (control)
1: Mild pleocellular infiltrates (A few scattered cells))
2: Moderate pleocellular infiltrate (between 10-30 cells per high magnification field (40x))
3: Severe pleocellular infiltrate (More than 30 cells per high magnification field)

17

Chapter 2

Sample Photomicrographs

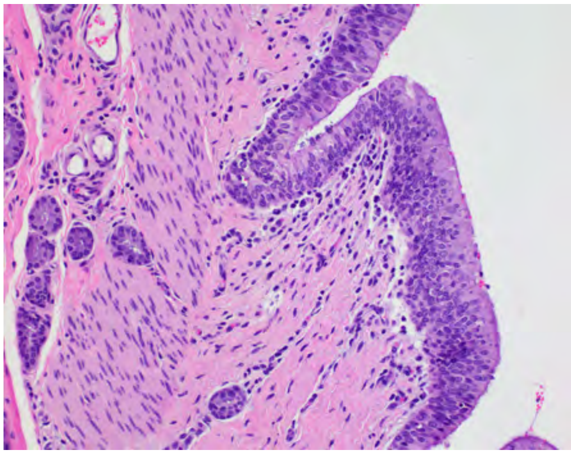

Figure 2.1: Normal bronchus 20X

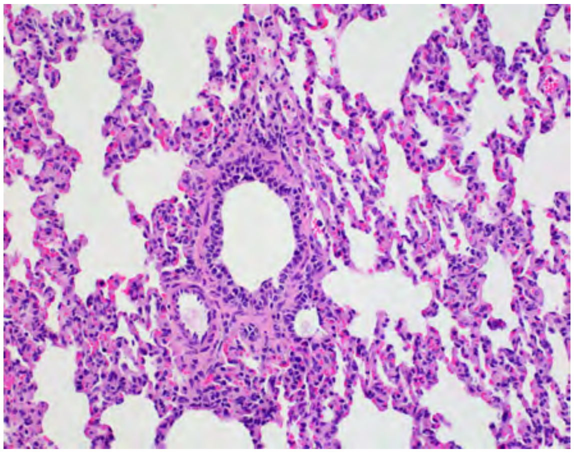

Figure 2.2: Normal bronchiole 20X

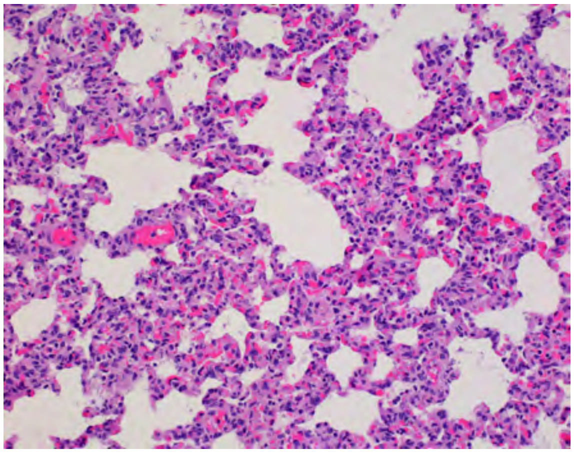

Figure 2.3: Normal alveolus 20X

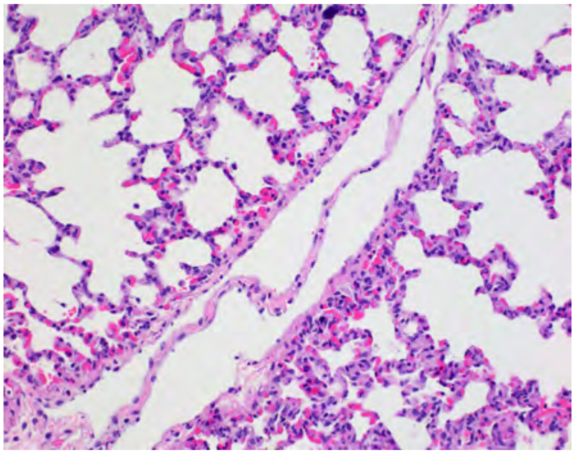

Figure 2.4: Normal pulmonary septum 20X

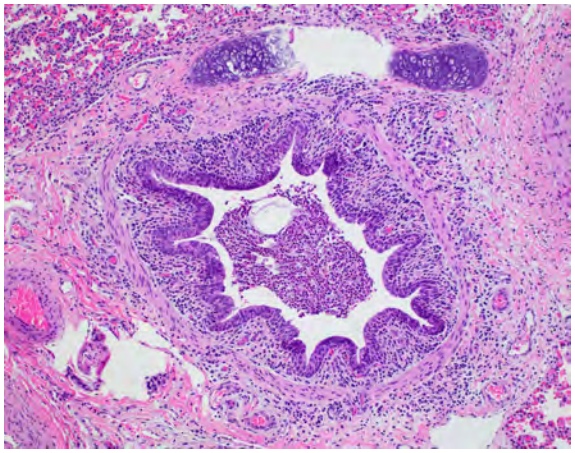

Figure 2.5: Neutrophilic exudate in bronchiolar lumen and peribronchiolar mononuclear infiltrates. 10X

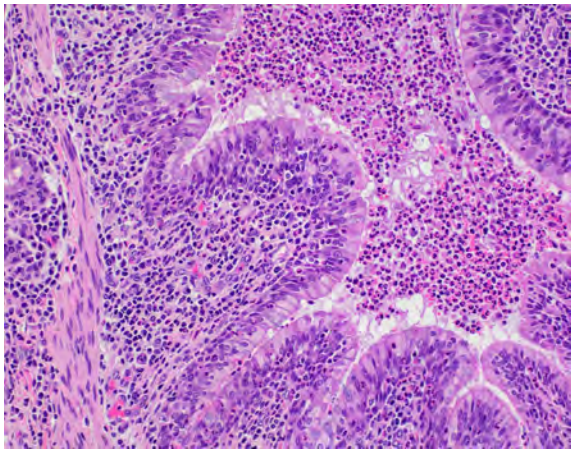

Figure 2.6: Neutrophilic exudate in the bronchial lumen, mononuclear infiltrates in bronchial wall, and multifocal transmigration of neutrophils in the bronchial mucosa. 20X

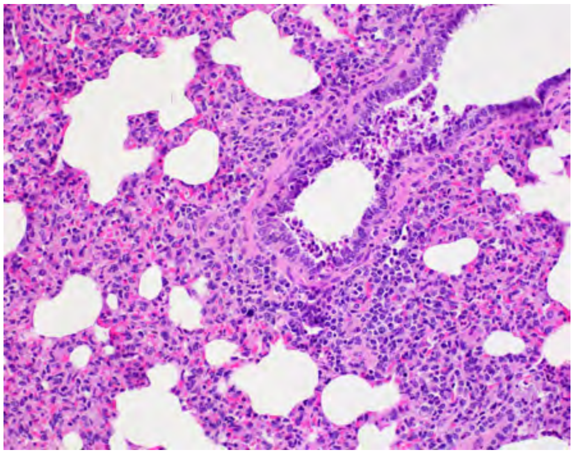

Figure 2.7: Small numbers of neutrophils in bronchiolar lumen , mild mononuclear infiltrate in the bronchiolar wall, and moderate thickening ig the alveolar septuae with mononuclear cells. 20X

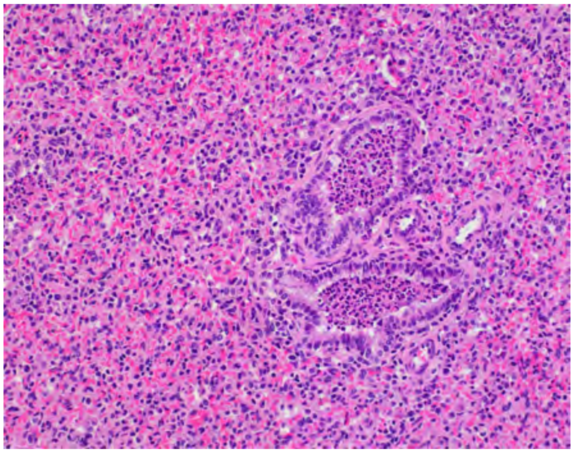

Figure 2.8: Neutrophilic exudate occluding a bronchiolar lumen and severe atelecatasis of adjacent pulmonary parenchyma. 20X

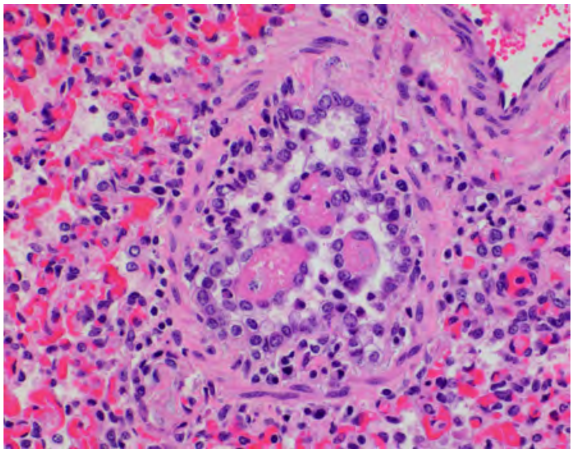

Figure 2.9: Early bronchiolitis obliterans 40X

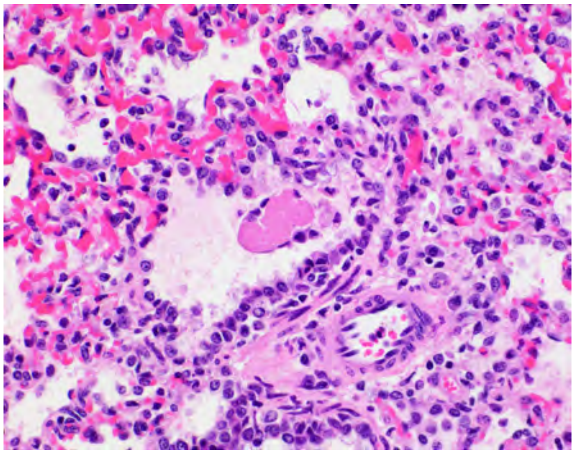

Figure 2.10: Within the bronchiolar lumen, there is a small deposit of fibrin, which is lined with a few epithelial cells and macrophages. This lesion was interpreted as early bronchiolitis obliterans. Hematoxylin and eosin, 40X.

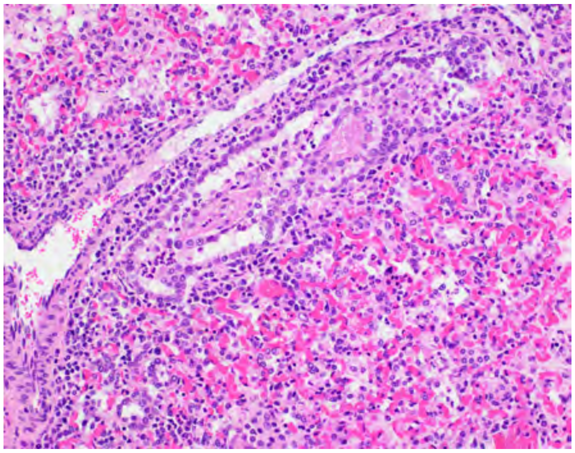

Figure 2.11: Bronchiolitis obliterans in the lumen of a bronchiole and mild hyperplasia of type II pneumocytes of the adjacent alveolar septum. 20X

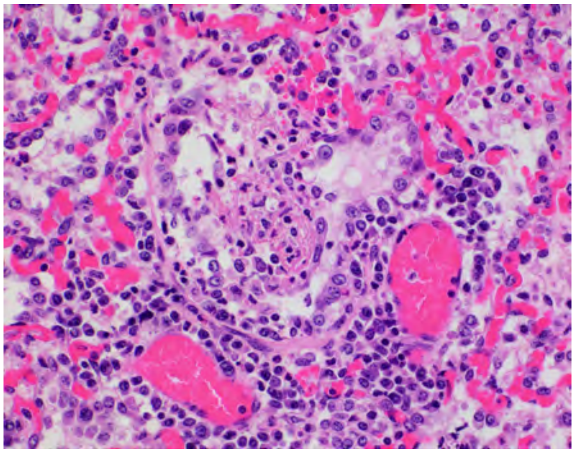

Figure 2.12: Bronchiolitis obliterans and mild peribronchiolar mononuclear infitrates. 40X

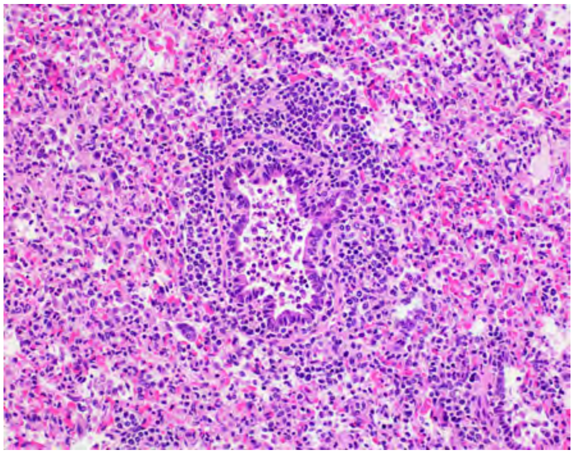

Figure 2.13: Intraluminal bronchiolar neutrophilic exudate, peribronchiolar mononuclear infiltrate and atelectasis of adjacent alveolar septum. 20X

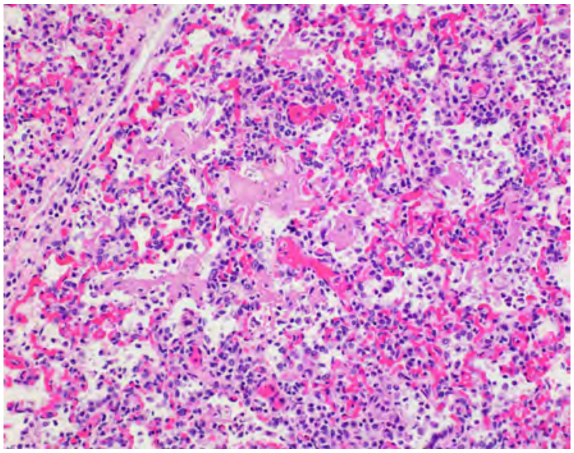

Figure 2.14: Fibrin and neutrophilic inifiltrates in in alveolar space. 20X

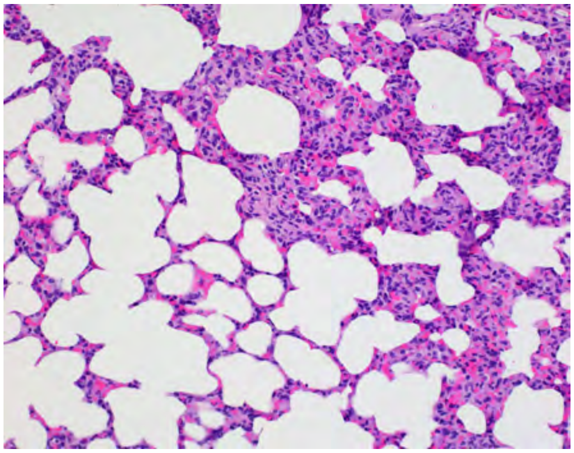

Figure 2.15: Mild thickening of alveolar septae (upper right) with mononuclear cells. 20X

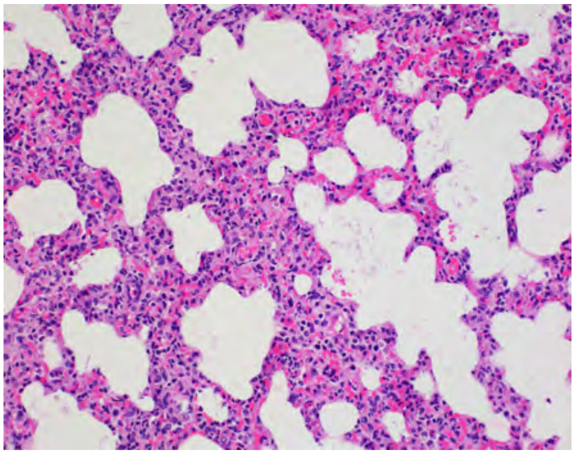

Figure 2.16: Moderate thickening of alveolar septae with mononuclear cells. 20X

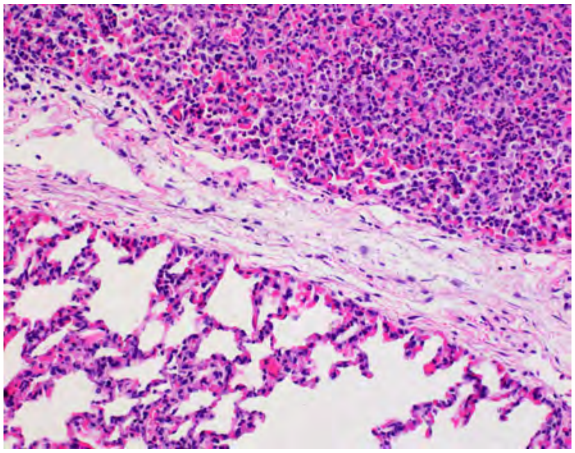

Figure 2.17: Mild expansion interlobular septum with edema, and atelectasis of pulmonary parenchyma, with small numbers of neutrophils (upper half). 20X

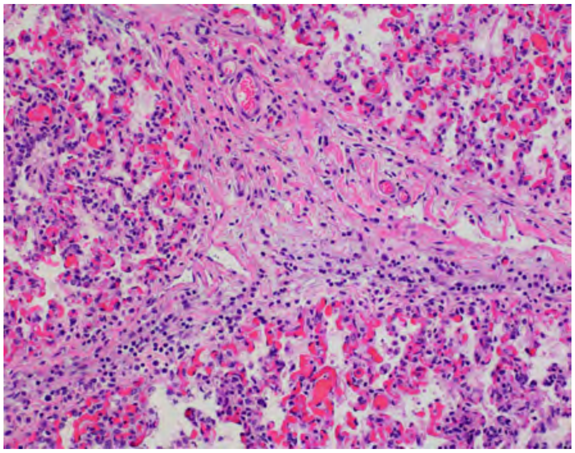

Figure 2.18: Mild expansion of the interlobular septum with mild inifitrates of mononuclear cells. 20X

Chapter 3

Data Entry

A sample data entry form printed in spreadsheet software is shown overleaf. This required extensive coding to normalize it and to make it usable for Stata. Ideally, a database rather than excel should be used for data entry tools. However, this tool has the advantage of being extremely efficient for the microscopist and is instantly readable for humans.

For subsequent code the first letter identifies where the sample was taken from, the second part the structure, the third part the specific finding e.g. ABronchussdeciliation refers to slide deciliation graded 0 to 3 in the bronchus from slide A.

See the manuscript and this early working paper see our how gross pathology was incorporated to deal with the discontinuous nature of lung pathology and validated with respect to clinical scores in life.

Microscopic analysis BRSV project

S1805648 D

		A	B	C	D	E	F	G	H	I	J	K	L
Bronchi	Neutrophilic exudate	1	0	2	1	1	0	1	0	0	0	0	0
	Deciliation	0	0	0	0	0	0	0	0	0	0	0	0
	Epithelial transmigration	1	0	1	0	0	0	1	1	0	0	0	0
	Intraepithelial pustules	0	0	0	0	0	0	0	0	0	0	0	0
	Inclusion bodies	0	0	0	0	0	0	0	0	0	0	0	0
	MN infiltrates submucosa	1	1	2	1	1	1	2	2	1	0	0	0
	Lymphoid nodules	1	0	1	0	0	0	0	0	0	0	0	0
Bronchioli	Neutrophilic exudate	0	0	2	1	1	0	1	2	1	0	0	0
	Fibrinous exudate	0	0	0	0	0	0	0	0	0	0	0	0
	Obliterating bronchiolitis	0	0	0	0	0	0	0	0	0	0	0	0
	necrosis of epithelium	0	0	0	0	0	0	0	0	0	0	0	0
	Inclusion bodies	0	0	0	0	0	0	0	0	0	0	0	0
	Epithelial transmigration	0	0	0	0	0	0	0	0	0	0	0	0
	Peribronchiolar mn infiltr	1	2	2	1	1	1	1	1	1	0	0	0
	Peribrionchiolar lym nod	0	0	0	0	0	0	0	0	0	0	0	0
Alveolus	Neutrophilic exudate	0	0	2	0	0	1	2	2	1	0	0	0
	Fibrinous exudate	0	0	0	0	0	0	0	0	0	0	0	0
	necrosis	0	0	0	0	0	0	0	0	0	0	0	0
	Edema	0	0	0	0	0	0	1	0	0	0	0	0
	Hemorrhages	0	0	0	0	0	0	0	0	0	0	0	0
	Syncitial cells	0	0	0	0	0	0	0	0	0	0	0	0
	Hyperpl type II pneumo	0	0	0	0	0	0	0	0	0	0	0	0
	Granulomas	0	0	0	0	0	0	0	0	0	0	0	0
	Atelectasis	0	0	2	0	1	0	2	2	1	0	0	0
Interstitium	Thickening mn	1	1	1	1	1	0	0	1	0	0	0	0
	Thickening pmn neu/eos	0	0	0	0	0	0	0	0	0	0	0	0
	vasculitis	0	0	0	0	0	0	0	0	0	0	0	0
	Thrombosis	0	0	0	0	0	0	0	0	0	0	0	0
	Lymphoid nodules	1	0	0	0	0	0	0	0	0	0	0	0
Septae	Expansion	2	1	1	0	1	1	2	2	2	1	1	1
	edema	1	1	1	0	1	1	2	2	2	1	1	1
	Fibrin	0	0	0	0	0	0	0	0	0	0	0	0
	Mn infiltrates	1	1	0	0	1	1	2	1	0	0	0	0
	PMN infiltrates	0	0	0	0	0	0	0	0	0	0	0	0
	Pleo infiltrates	0	0	0	0	0	0	0	0	0	0	0	0
	Arteritis	0	0	0	0	0	0	0	0	0	0	0	0
Pleura	Thickening	1	1	0	0	0	1	0	0	0	0	0	0
	Fibrosis	2	1	0	0	0	0	0	0	0	0	0	0
	Lymphatic dilation / edema	1	1	0	0	0	1	0	0	0	0	0	0
	MN infiltrates	0	1	0	0	0	1	0	0	0	0	0	0
	PMN infiltrates	0	0	0	0	0	0	0	0	0	0	0	0
	Pleo infiltrates	0	0	0	0	0	0	0	0	0	0	0	0

Other findings

Trachea	Deciliation	
	Epithelial transmigration	
	MN infiltrates submucosa	
	Lymphoid nodules	

Bronchial lymph node	Hyperplasia/reactive	
	Serocellular sinus exudate	
	Mn cell infiltrates (cap/adv)	
	PMN infiltrates (cap/adv)	
	Pleo infiltrates (cap/adv)	

Chapter 4

Plotting the results using Matrix plots

4.1 Statistical code for Matrix plots

This was performed in Stata 16.1 but will run under many earlier versions alos. It can be future proffed using version control in the do file.

Steps

Download Adrian Mander′ s (MRC Biostatistics Unit, Cambridge, UK.) plotmatrix.ado file from the Stata SSC archive.

```
ssc install plotmatrix
```

Set the sort order to allow subsequent labelling of individual calves.

```
sort drug_treat replicate tag
```

Create a matrix of the variables to be plotted:

```
mkmat   *_Bronchioli* qmax_collie qmean_collie
   qmazviral_load qmeanviral_load ,mat(bronchioli)
```

Plot the matrixplots

```
plotmatrix  ,mat(bronchioli)  split(0 1 2 3 4 )
   maxticks(198)
 xlab(,labsize(tiny)) xlab(,angle(270)) legend(off)
  yline( -5.5 -11.5 -17.5 -23.5 -29.5 ,
  lwidth(thin) lcolor(red) lstyle(foreground)) ylab
     (,angle(0)
  labsize(vsmall))  legend(size(small)) scheme(
     Plotplainblind)
   title(Bronchiolus histology findings)
```

Additional editing was performed in the Stata graph editor and recorded in the .grec files. These .grec can be edited manually but are verbose and typically re-run as saved via the editor.

```
gr play bronchioles_relable.grec
```

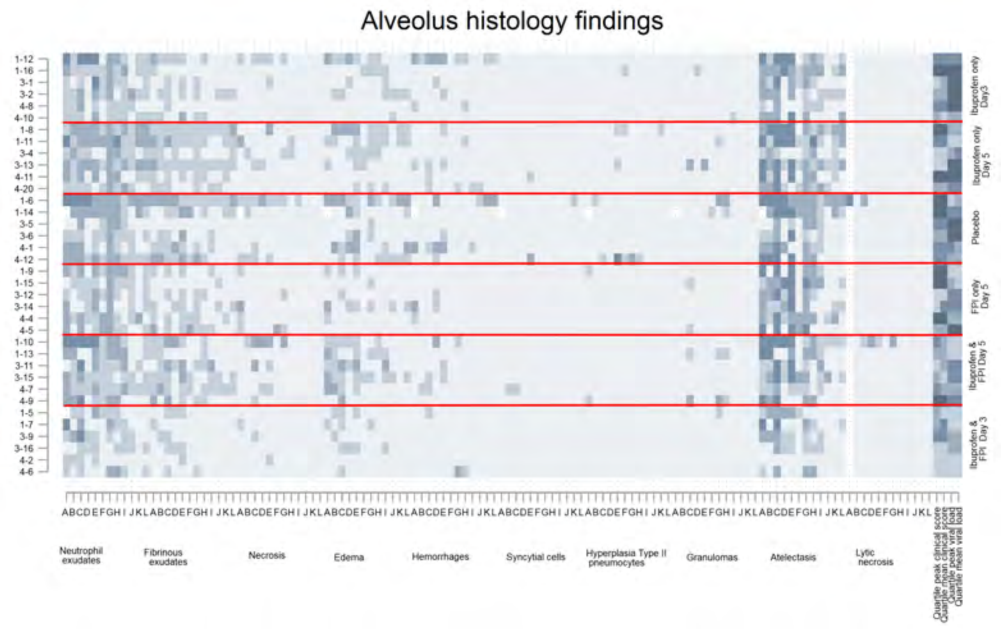

Chapter 5

Performing Canonical Discrimination

5.1 Statistical code needed to perform canonical discriminant analysis

This summarizes the code. This code was run under Stata 16.1 but will likely work under most recent earlier versions. It will run in future versions by inserting the command.

```
version 16.1
```

The following code is unique to this study. It creates and applies labels for subsequent output and converts the some microscopists or data entry clerks habit of leaving normal as blank or placing a check mark instead of writing 0. Here we assume the use of consistent slide nomenclature throughout the analysis as we have done with a diagram of the lungs showing from where each slide is to be taken.

```
 //////Interstitium

foreach v in "A" "B" "C" "D" "E" "F" "G" "H" "I"  "J"
   "K" {

foreach var of varlist  `v'_Bronchi_Deciliation␣`v'
   _Bronchi_Epitheltransmigrat
`v'_Bronchi_Inclusionbodies␣`v'
   _Bronchi_Intraepithelialpust
`v'_Bronchi_Lymphoidnodules
`v'_Bronchi_MNinfilsubmucosa `v'
   _Bronchi_Neutrophilexud␣{
```

```
␣␣␣␣␣␣␣␣
␣␣␣␣␣␣␣␣replace␣`var' =0 if `var'␣==.␣␣␣
}
```

The code below checks for collinearity. The analyst then has to iteratively drop collinear variables. When choosing between variables the analyst will need to rely on domain knowledge. To ensure you are not modelling noise, create a random variable for your dataset and use this as the grouping variable and visually compare the results with what you are getting with the study variable.

```
 _rmcoll   `v'_Bronchi_Deciliation␣`v'
    _Bronchi_Epitheltransmigrat
 `v'_Bronchi_Inclusionbodies
␣`v'_Bronchi_Intraepithelialpust `v'
   _Bronchi_Lymphoidnodules
␣`v'_Bronchi_MNinfilsubmucosa
  `v'_Bronchi_Neutrophilexud
}
```

To look at a single slide across all animals study one would use:

```
 candisc   A_Bronchi_Epitheltransmigrat
    A_Bronchi_Intraepithelialpust
 A_Bronchi_Lymphoidnodules
 A_Bronchi_MNinfilsubmucosa A_Bronchi_Neutrophilexud
 if died ==1 ,gr(drug_treat)
```

followed by

```
loadingplot
```

and

```
scoreplot
```

5.1.1 Improving the graphs

The actual graphs for publication need to be created differently to allow for improved clarity, color etc. A sample of the actual code used for this appears below:

```
#delimit ;

candisc
A_Pleura_Fibrosis A_Pleura_Lymphaticdilation
A_Pleura_Lymphaticdilation_edema
A_Pleura_MNinfil   A_Pleura_Pleoinfil
 B_Pleura_Fibrosis
```

```
 B_Pleura_Lymphaticdilation_edema
 B_Pleura_MNinfil
 C_Pleura_Fibrosis C_Pleura_Lymphaticdilation
 C_Pleura_Lymphaticdilation_edema
 C_Pleura_MNinfil
 D_Pleura_Fibrosis D_Pleura_Lymphaticdilation
  D_Pleura_Lymphaticdilation_edema
 D_Pleura_MNinfil
 E_Pleura_Lymphaticdilation
 E_Pleura_Lymphaticdilation_edema
 E_Pleura_Pleoinfil
 F_Pleura_Lymphaticdilation_edema F_Pleura_MNinfil
 G_Pleura_Lymphaticdilation_edema G_Pleura_MNinfil
 H_Pleura_Lymphaticdilation
    H_Pleura_Lymphaticdilation_edema
 H_Pleura_MNinfil
 I_Pleura_Lymphaticdilation_edema I_Pleura_MNinfil
 J_Pleura_Fibrosis
 K_Pleura_Lymphaticdilation_edema

 if died==1 ,gr(drug_treat)

;
#delimit cr

  loc v1 drug_treat
loc v2 "scoreplot_label"
loc size "tiny"
loc title2 "Pleura␣Histology␣"
loc msize "small"
loc angle =45
loc angle1 =0
loc angle2 =90

cap drop  scoreF1 scoreF2
predict scoreF1 scoreF2  ,dscore
#delimit ;

tw
 (sc scoreF2 scoreF1  if `v1'␣==1␣,mlabel()␣␣mlabsize
    ("`size'")␣msize("`msize'")
␣␣␣msymbol(oh)␣mlabangle(`angle'))
 (sc scoreF2 scoreF1  if `v1'␣==2,␣mlabel()␣mlabsize
    ("`size'")␣msize("`msize'")
␣␣␣msymbol(o)␣mlabangle(`angle'))
```

```
 (sc scoreF2 scoreF1  if `v1'␣==3,␣mlabel()␣mlabsize
    ("`size'")␣msize("`msize'")
␣␣␣msymbol(O)␣mlabangle(`angle2'))
 (sc scoreF2 scoreF1  if `v1'␣==4,␣␣mlabel()␣mlabsize
    ("`size'")␣msize("`msize'")
␣␣␣msymbol(s)␣mlabangle(`angle1'))
 (sc scoreF2 scoreF1  if `v1'␣==5,mlabel()␣mlabsize("`
    size'")␣msize("`msize'")
␣␣␣mlabangle(`angle'))
 (sc scoreF2 scoreF1  if `v1'␣==6␣,
␣␣␣␣␣␣␣␣␣␣␣␣␣␣␣␣title("Score␣plot␣of␣`title2' and drug
   "
␣␣␣␣␣␣␣␣␣␣␣␣␣␣␣␣␣"Discriminant function scores"␣,size(
   small)␣)
␣␣␣␣␣␣␣␣␣␣␣␣␣␣␣␣␣␣yline(0)␣xline(0)␣xtitle(
   Discriminant␣score␣1,size(vsmall))
␣␣␣␣␣␣␣␣␣␣␣␣␣␣␣␣␣␣ytitle(Discriminant␣score␣2␣,size(
   vsmall))
␣␣␣␣␣␣␣␣␣␣␣␣␣␣legend(on)␣␣mlabel()␣msize("`msize'")
␣␣␣␣␣␣␣␣␣␣␣␣␣␣␣mlabsize("`size'")␣msymbol(S)␣mlabangle
   (`angle1')
␣␣␣␣␣␣␣␣␣␣␣␣␣␣␣xlabel(,labsize(vsmall))
␣␣␣␣␣␣␣␣␣␣␣␣␣␣␣ylabel(,labsize(vsmall))
␣␣␣␣␣␣␣␣␣␣␣␣␣␣␣␣scheme(plotplainblind))
;

#delimit␣cr

␣gr␣save␣"`title2'"␣,replace
```

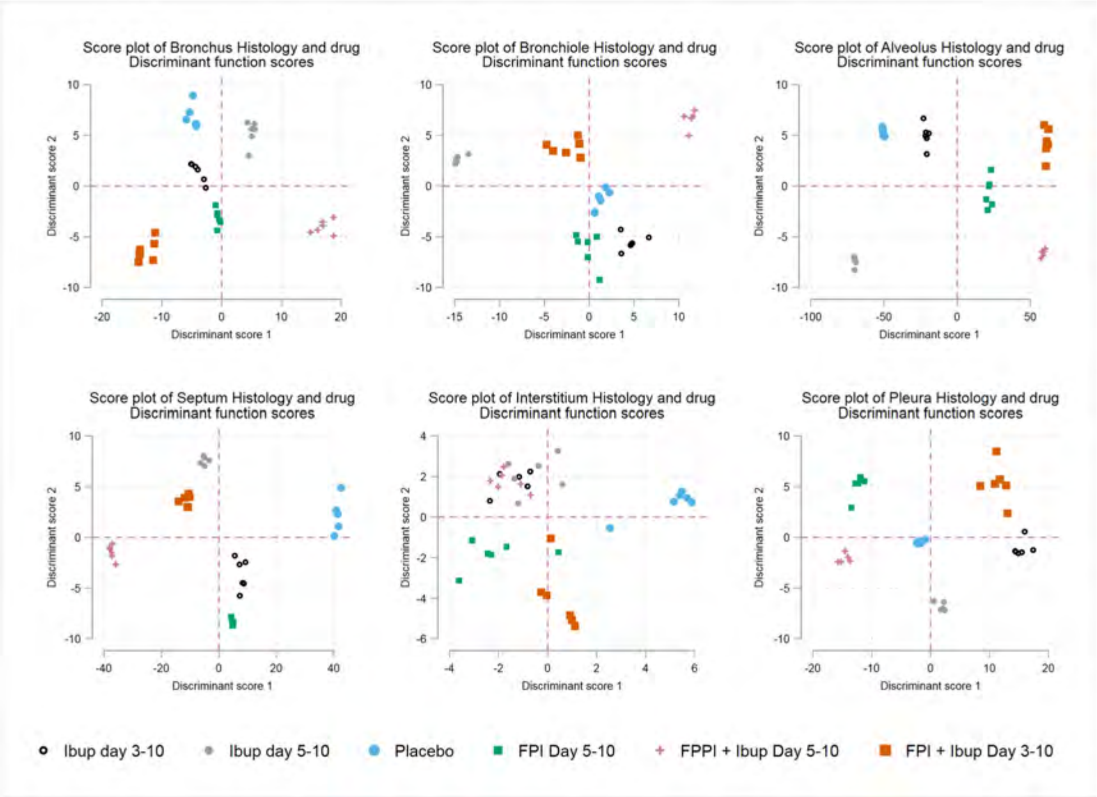

Figure 5.1: Canonical dicriminanat analysis plot with labeling.
