# Supplementary material for: A randomized controlled trial comparing non-steroidal anti-inflammatory and fusion protein inhibitors singly and in combination on the histopathology of bovine respiratory syncytial virus infection
Source: PLoS One. 2021 Jun 10;16(6):e0252455. doi: 10.1371/journal.pone.0252455 (PMC8191941; doi:10.1371/journal.pone.0252455)
Supplement: S2 File — (PDF) [file pone.0252455.s002.pdf]

Replicate	tag	Bronchial_swa	bronchi	Lung	BRSV	BVD1	IBR	Coronavirus
1	6	Strep gallolyticu	Mycoplasma	Mycoplasma	1	0	0	0
1	10	0	Mycoplasma	Mycoplasma	Indeterminat	0	0	0
1	7	0	Mycoplasma	Mycoplasma	Indeterminat	0	0	0
1	8	Klebsiella oxyto	Klebsiella oxytoca, Mycoplasma	Klebsiella oxytoca, mycopla	Indeterminat	0	0	0
1	16	Strep ssp, pastu	Mycoplasma	Mycoplasma	1	1	0	0
1	9	Strep gallolyticu	Mycoplasma	Mycoplasma	Indeterminat	0	0	1
1	11	Strep ssp,	Mycoplasma	Trueperella pyogenes, Stre	Indeterminat	0	0	0
1	12	0	Mycoplasma	Mycoplasma	0	0	0	0
1	5	0	Mycoplasma	mycoplasma, strep sp	0	0	0	0
1	15	Mannheimia rar	Mycoplasma	Mycoplasma, Mannheim1ia	1	Indeterminat	0	0
1	14	Trueperella	Mycoplasma	Trueperella pyogenes, , My	1	Indeterminat	0	0
1	13	0	Mycoplasma	Mycoplasma	Indeterminat	Indeterminat	0	0
3	1	0	0	0	1	Indeterminat	0	0
3	2	Staphylococcus	0	0	Indeterminat	0	0	0
3	4	0	0	0	1	0	0	0
3	5	0	0	0	Indeterminat	Indeterminat	0	0
3	6	0	0	Acinetobacter lwoffi Rare# A	1	0	0	0
3	9	Streptococcus s	0	0	1	Indeterminat	0	0
3	11	Streptococcus s	0	0	Indeterminat	0	0	0
3	12	0	0	0	Indeterminat	0	0	0
3	13	0	0	0	Indeterminat	Indeterminat	0	0
3	14	0	0	0	1	0	0	0
3	15	0	0	0	Indeterminat	0	0	0
3	16	0	0	0	Indeterminat	Indeterminat	0	0
4	5	0	0	0	1	0	0	0
4	6	Mixed flora	0	0	1	0	0	0
4	8	Mixed flora	0	0	1	0	0	0
4	9	Mixed flora	0	0	Indeterminat	0	0	0
4	12	0	0	0	Indeterminat	0	0	0
4	20	Mixed flora	0	0	Indeterminat	0	0	0
4	11	Strep gallolyticus		Mycoplasma ssp not bovis	1	0	0	0
4	1	Strep gallolyticus, mixed flora		Mycoplasma ssp not bovis	1	0	0	0
4	2	Strep gallolyticus		0	Indeterminat	0	0	0
4	4	Trueperella pyogenes, kocuriarhizophila		mixed flora, mycoplasma	1	0	0	0
4	7	Strep gallolyticus		Mycoplasma ssp not bovis	Indeterminat	0	0	0
4	10	0		corynbacteria, mixed flora,	1	0	0	0

1 =Detected, 0 Not detected
