# Supplementary material for: A randomized controlled trial comparing non-steroidal anti-inflammatory and fusion protein inhibitors singly and in combination on the histopathology of bovine respiratory syncytial virus infection
Source: PLoS One. 2021 Jun 10;16(6):e0252455. doi: 10.1371/journal.pone.0252455 (PMC8191941; doi:10.1371/journal.pone.0252455)
Supplement: S3 File — (DOC) [file pone.0252455.s003.doc]

S3 File. Statistical code for Matrix plots

This was performed in Stata 16.1.

Steps

Download

Adrian Mander’s (MRC Biostatistics Unit, Cambridge, UK.) plotmatrix.ado file from the Stata SSC archive.

ssc install plotmatrix

Set the sort order to allow subsequent labelling of individual calves.

sort drug_treat replicate tag

Create a matrix of the variables to be plotted:

mkmat *_Bronchioli* qmax_collie qmean_collie qmazviral_load qmeanviral_load,mat(bronchioli)

Plot the matrixplots

plotmatrix ,mat(bronchioli) split(0 1 2 3 4 ) maxticks(198) xlab(,labsize(tiny)) xlab(,angle(270)) legend(off) yline( -5.5 -11.5 -17.5 -23.5 -29.5 ,lwidth(thin) lcolor(red) lstyle(foreground)) ylab(,angle(0) labsize(vsmall)) legend(size(small)) scheme(Plotplainblind) title(Bronchiolus histology findings)

Additional editing was performed in the Stata graph editor and recorded in the .grec files.

gr play bronchioles_relable.grec
