# Supplementary material for: A randomized controlled trial comparing non-steroidal anti-inflammatory and fusion protein inhibitors singly and in combination on the histopathology of bovine respiratory syncytial virus infection
Source: PLoS One. 2021 Jun 10;16(6):e0252455. doi: 10.1371/journal.pone.0252455 (PMC8191941; doi:10.1371/journal.pone.0252455)
Supplement: S4 File — (DOC) [file pone.0252455.s004.doc]

S4 File. Statistical code needed to perform canonical discriminant analysis

This code was run under Stata 16.1 but will likely work under most recent earlier versions. It will run in future versions by inserting the command

version 16.1

The following code is unique to this study. It creates and applies labels for subsequent output and converts the microscopist’s habit of leaving normal as blank instead of writing 0.

// Labels for scoreplots

cap lab drop scoreplot_label

lab def scoreplot_label 1 "Ibup Day 3-10" 2 "Ibup Day 5-10" 3 "Placebo x2" 4 "FPI Day 5-10" 5 "FPI + Ibup Day 5-10" 6 "FPI + Ibup Day 3 -10"

//////Interstitium

foreach v in "A" "B" "C" "D" "E" "F" "G" "H" "I" "J" "K" {

foreach var of varlist `v'_Bronchi_Deciliation `v'_Bronchi_Epitheltransmigrat `v'_Bronchi_Inclusionbodies `v'_Bronchi_Intraepithelialpust `v'_Bronchi_Lymphoidnodules `v'_Bronchi_MNinfilsubmucosa `v'_Bronchi_Neutrophilexud {

replace `var' =0 if `var' ==.

}

The code below checks for collinearity. The analyst then has to iteratively drop collinear variables. When choosing between variables the analyst will need to rely on domain knowledge. To ensure you aree not modelling noise, create a random variable for your dataset and use this as the grouping variable and visually compare the results with what you are getting with the study variable.

_rmcoll `v'_Bronchi_Deciliation `v'_Bronchi_Epitheltransmigrat `v'_Bronchi_Inclusionbodies `v'_Bronchi_Intraepithelialpust `v'_Bronchi_Lymphoidnodules `v'_Bronchi_MNinfilsubmucosa `v'_Bronchi_Neutrophilexud

}

To look at a single slide across all animals study one would use:

candisc A_Bronchi_Epitheltransmigrat A_Bronchi_Intraepithelialpust A_Bronchi_Lymphoidnodules A_Bronchi_MNinfilsubmucosa A_Bronchi_Neutrophilexud if died ==1 ,gr(drug_treat)

followed by

loadingplot

and

scoreplot

The actual graphs for publication need to be created differently to allow for improved clarity, color etc. A sample of the actual code used for this appears below:

#delimit ;

candisc

A_Pleura_Fibrosis A_Pleura_Lymphaticdilation A_Pleura_Lymphaticdilation_edema A_Pleura_MNinfil A_Pleura_Pleoinfil B_Pleura_Fibrosis

B_Pleura_Lymphaticdilation_edema B_Pleura_MNinfil

C_Pleura_Fibrosis C_Pleura_Lymphaticdilation C_Pleura_Lymphaticdilation_edema C_Pleura_MNinfil

D_Pleura_Fibrosis D_Pleura_Lymphaticdilation D_Pleura_Lymphaticdilation_edema D_Pleura_MNinfil

E_Pleura_Lymphaticdilation E_Pleura_Lymphaticdilation_edema E_Pleura_Pleoinfil

F_Pleura_Lymphaticdilation_edema F_Pleura_MNinfil

G_Pleura_Lymphaticdilation_edema G_Pleura_MNinfil

H_Pleura_Lymphaticdilation H_Pleura_Lymphaticdilation_edema H_Pleura_MNinfil

I_Pleura_Lymphaticdilation_edema I_Pleura_MNinfil

J_Pleura_Fibrosis

K_Pleura_Lymphaticdilation_edema

if died==1 ,gr(drug_treat)

;

#delimit cr

loc v1 drug_treat

loc v2 "scoreplot_label"

loc size "tiny"

loc title2 "Pleura Histology "

loc msize "small"

loc angle =45

loc angle1 =0

loc angle2 =90

cap drop scoreF1 scoreF2

predict scoreF1 scoreF2 ,dscore

#delimit ;

tw

(sc scoreF2 scoreF1 if `v1' ==1 ,mlabel() mlabsize("`size'") msize("`msize'") msymbol(oh) mlabangle(`angle'))

(sc scoreF2 scoreF1 if `v1' ==2, mlabel() mlabsize("`size'") msize("`msize'") msymbol(o) mlabangle(`angle'))

(sc scoreF2 scoreF1 if `v1' ==3, mlabel() mlabsize("`size'") msize("`msize'") msymbol(O) mlabangle(`angle2'))

(sc scoreF2 scoreF1 if `v1' ==4, mlabel() mlabsize("`size'") msize("`msize'") msymbol(s) mlabangle(`angle1'))

(sc scoreF2 scoreF1 if `v1' ==5,mlabel() mlabsize("`size'") msize("`msize'") mlabangle(`angle'))

(sc scoreF2 scoreF1 if `v1' ==6 ,

title("Score plot of `title2' and drug" "Discriminant function scores" ,size(small) ) yline(0) xline(0) xtitle(Discriminant score 1,size(vsmall)) ytitle(Discriminant score 2 ,size(vsmall))

legend(on) mlabel() msize("`msize'") mlabsize("`size'") msymbol(S) mlabangle(`angle1') xlabel(,labsize(vsmall))

ylabel(,labsize(vsmall)) scheme(plotplainblind))

;

#delimit cr

gr save "`title2'" ,replace
